# Association between neuroticism and physical activity: a systematic review and meta-analysis

**DOI:** 10.3389/fnhum.2025.1557739

**Published:** 2025-09-25

**Authors:** Wenxue Ma, Xiaotian Wang, Wentao Qiu, Yuyang Nie, Rong Gao, Cong Liu

**Affiliations:** ^1^College of Physical Education and Sports, Beijing Normal University, Beijing, China; ^2^College of Education for the Future, Beijing Normal University, Zhuhai, China

**Keywords:** neuroticism, personality, physical activity, correlation, influencing factors, potential psychological mechanisms

## Abstract

**Background:**

Physical activity has been shown to be associated with neuroticism, a personality trait reflecting emotional instability and a tendency toward negative emotions. Understanding this relationship is crucial for developing effective mental health interventions. However, the underlying mechanisms and the strength of this association remain insufficiently understood.

**Objective:**

This systematic review and meta-analysis aims to examine the current research on the relationship between neuroticism and physical activity, analyze their correlations and moderating factors, and investigate the potential bidirectional mechanisms linking these two factors.

**Methods:**

Following the PRISMA guidelines, we conducted a comprehensive search of Web of Science, PubMed, ProQuest, Scopus, and EBSCOhost for studies published between January 2000 and November 2024. We included English-language studies across all age groups that employed cross-sectional, longitudinal, or cohort designs. Studies focusing on special populations, non-peer-reviewed works, samples with fewer than 50 participants, non-empirical studies, and reviews were excluded. Data extraction was performed using standardized forms, and a meta-analysis was conducted in Stata 18 to assess heterogeneity and publication bias.

**Results:**

After screening, 25 studies were included, comprising 15 Pearson correlation analyses and 12 multiple regression analyses. The meta-analysis revealed a significant negative correlation between physical activity and neuroticism, with an average correlation coefficient *r* = −0.141. This suggests that higher levels of physical activity are associated with lower levels of neuroticism. Specifically, the average standardized coefficient β for neuroticism inhibiting physical activity was −0.150, indicating that for every one standard deviation increase in neuroticism, physical activity decreases by approximately 0.150 standard deviation units. Conversely, the average standardized coefficient β for physical activity affecting neuroticism was −0.113, suggesting a potential reduction in neuroticism with increased physical activity, although this effect was not statistically significant across the limited number of studies.

**Conclusion:**

Our findings confirm a significant negative association between physical activity and neuroticism, highlighting the potential of physical activity as a strategy for improving mental health. However, establishing causality requires further verification through longitudinal and experimental designs. The results emphasize the need for personalized interventions targeting individuals with high neuroticism. Future research should prioritize diverse cultural samples, standardized measurement protocols, and mechanistic investigations of this bidirectional relationship to better understand the underlying processes and develop effective interventions.

**Systematic review registration:**

https://www.crd.york.ac.uk/PROSPERO/view/CRD420251051360, identifier: CRD420251051360.

## Introduction

Personality traits are internal neuropsychological structures that influence individual behavior (McCrae and Costa, 1997). According to Simon et al. (2020), these traits represent consistent behavioral tendencies displayed by individuals across various contexts. Personality traits are effective predictors of individual behaviors and serve as important markers for distinguishing between individuals. The Five-Factor Model (FFM) is a widely recognized theoretical framework for personality traits, encompassing five dimensions: Neuroticism, Extraversion, Openness, Agreeableness, and Conscientiousness. Neuroticism reflects an individual's emotional stability and tendency toward negative emotions (Laursen et al., 2010). Extraversion indicates the extent of social interaction (Mesurado et al., 2014). Openness represents creativity and curiosity (Gjerde and Cardilla, 2009). Agreeableness reflects interpersonal attitudes, while Conscientiousness reflects caution and persistence in goal-directed behaviors (De Fruyt et al., 2015).

Neuroticism is a key personality trait associated with various physical and mental health conditions and comorbidities. It can predict these conditions and is linked to healthcare and mental health service utilization (Widiger and Oltmanns, 2017). Individuals high in neuroticism often struggle with stress management and frequently experience feelings of threat, overwhelm, and desperation in daily life (Lahey, 2009). Lower neuroticism correlates with increased physical activity and reduced sedentary behavior (Sutin et al., 2016). Higher neuroticism is also associated with cognitive decline and poor cognitive performance. Regular physical activity across life stages can enhance cognitive or brain reserve, enabling the brain to maintain resilience despite cognitive obstacles or pathologies (Arida and Teixeira-Machado, 2021). Individuals with lower neuroticism appear to exhibit greater pathological resilience or stronger cognitive function, even in the presence of potential neuropathological issues (Graham et al., 2021).

Physical activity is a critical factor for mental health and increases energy expenditure (Bull et al., 2020). Exercise, defined as bodily activity with specific intensity, frequency, and duration, aims to improve physical health (Liu, 2020). Research by Qiu et al. (2025a) and Qiu et al. (2025b) demonstrates that physical activity can alleviate anxiety. However, surveys indicate that both adults and adolescents often fail to meet recommended physical activity levels for health promotion (Harris et al., 2013; Physical Activity Guidelines Advisory Committee, 2008). A study across 15 European countries also highlights insufficient physical activity participation (Ruiz et al., 2011). Reduced physical activity is associated with unhealthy lifestyles and psychological issues (Brunet et al., 2013). This public health issue has garnered widespread attention, yet current interventions often yield limited success (Heath et al., 2012; Kriemler et al., 2011; Metcalf et al., 2012).

Personality traits may influence physical activity levels, thereby affecting intervention efficacy and health outcomes (Bouchard et al., 2012). Theoretically, personality can account for inherent differences in physical activity engagement (Eysenck and Eysenck, 1985; Gray, 1991; Costa and McCrae, 1999). While studies have examined the associations between Extraversion, Conscientiousness, and physical activity, the role of Neuroticism in this context is particularly significant. High levels of Neuroticism are linked to lower physical activity participation, potentially due to heightened emotional instability and reduced motivation (Widiger and Oltmanns, 2017). This relationship is crucial to investigate, as addressing Neuroticism could enhance the effectiveness of interventions aimed at increasing physical activity and improving mental health.

Existing studies suggest that physical activity can positively influence traits such as Extraversion and Conscientiousness. For example, Lai and Qin (2018) found a positive association between Extraversion and physical activity levels, noting that outgoing individuals often engage in higher-intensity, socially demanding exercises. Mõttus et al. (2017) also observed a significant positive correlation between physical activity and Extraversion and Conscientiousness. A literature review and meta-analysis confirmed the significance of Extraversion, Neuroticism, and Conscientiousness as factors related to physical activity (Wilson and Dishman, 2015). Another meta-analysis showed that insufficient physical activity correlates with higher Neuroticism and lower Conscientiousness (Allen et al., 2017).

Physical exercise significantly impacts adolescent personality development, positively affecting trait stability (Rhodes and Smith, 2006). This suggests that interventions targeting physical activity could not only improve mental health but also contribute to positive personality development. However, the mechanisms linking Neuroticism to physical activity participation remain unclear. Understanding these mechanisms is essential for developing effective interventions that address the unique challenges faced by individuals with high Neuroticism.

Previous research has predominantly focused on specific populations, such as athletes or individuals with particular diseases, with limited attention to the general population (Bäckmand et al., 2003; Piepiora, 2021). This gap in the literature highlights the necessity to comprehensively explore the relationship between neuroticism and physical activity across diverse populations. Understanding this relationship will facilitate the development of tailored intervention strategies to address the unique challenges faced by individuals with high neuroticism, ultimately promoting greater participation in physical activity and improving psychological health outcomes.

In public health, the relationship between neuroticism and physical activity is highly significant. Presently, there is a scarcity of scholarly reviews on this topic, and the mechanisms linking neuroticism to physical activity participation remain unclear. Through a systematic review and meta-analysis, this study aims to comprehensively evaluate the overall correlation between neuroticism and physical activity and to explore whether this relationship is influenced by sample characteristics and study features. Additionally, the study will systematically review existing research, summarize and interpret relevant data, and analyze the underlying mechanisms. This approach will enhance our understanding of how neuroticism relates to physical activity and provide valuable theoretical support and practical guidance for improving individual health and promoting wellbeing. In turn, this will contribute to the advancement of related fields.

## Methods

### Literature search strategy

This study adhered to the standards proposed by the “Preferred Reporting Items for Systematic Reviews and Meta-Analyses (PRISMA) (Zhang et al., 2020)” statement, implementing a structured electronic literature search process. Databases including Web of Science, PubMed, ProQuest, Scopus, and EBSCO were searched. The search criteria were set to include documents where the title or abstract contained “physical activity” or “exercise” or “leisure-time activity” or “sport participation,” and simultaneously included “nervousness” or “neuroticism” or “neurotic personality.” Only peer-reviewed journal articles published in English were considered for this study. The literature search covered a time span from January 1, 2000, to November 20, 2024.

### Inclusion and exclusion criteria

Inclusion criteria were as follows: ①The study population encompassed all age groups;② The research must include an assessment of neuroticism;③ An evaluation of participants' physical activity was required;④ Studies should have conducted a quantitative analysis of the association between neuroticism and physical activity;? Study designs were cross-sectional, longitudinal, or long-term follow-up;? The literature was published in English.

Exclusion criteria included:① Studies focused on specific populations, such as individuals with disabilities, athletes, etc.;② Articles not published in peer-reviewed journals;③ Studies with a sample size of fewer than 50 participants;④ Research that did not provide data on the association between neuroticism and physical activity;? Review articles or those that employed regression analysis.

We prioritized studies with the highest sample independence from the same research team using overlapping samples. For studies using the same sample to explore different dimensions (e.g., accelerometer vs. self-report data), we pooled effect sizes to prevent duplication. If multiple studies used the same dataset with highly overlapping goals (e.g., only adjusting the statistical model), we included the one with more comprehensive methodology or complete effect sizes.

After deduplication of the retrieved literature, two researchers independently screened the articles based on predefined inclusion and exclusion criteria. Initially, titles and abstracts were reviewed to identify potentially relevant studies for full-text examination. In addition to incorporating studies confirmed through full-text review, we also conducted a thorough examination of the reference lists of the retrieved full-text articles and other systematic reviews to ensure that no eligible studies were overlooked. The final selection of included articles was determined through joint verification by the two researchers. In cases of inconsistent screening results, a third researcher was consulted to make the final decision.

### Rationale for exclusion criteria

Exclusion of Non-English Literature and Small Sample Studies: We acknowledge that excluding non-English literature and studies with a sample size of fewer than 50 participants may introduce selection bias. However, these criteria were chosen to ensure the highest quality and relevance of the studies included in our meta-analysis. Non-English literature was excluded to maintain consistency and to avoid potential translation errors and difficulties in obtaining full texts. Studies with small sample sizes were excluded to ensure robustness and reliability of the findings, as smaller samples may not provide sufficient statistical power to detect significant associations. We have added a discussion on these potential biases in the limitations section to highlight their potential impact on our findings.

Exclusion of Athletes and Special Populations: We excluded studies focused on athletes and other specific populations to ensure that our findings are generalizable to the broader population. While athletes and individuals with specific health conditions may have unique mechanisms linking neuroticism and physical activity, the general population is more diverse and less likely to engage in structured, high-intensity physical activity regimens. By focusing on the general population, we aim to provide insights that are applicable to a wider range of individuals. However, we recognize the importance of studying these specific populations and suggest that future research should explore the unique mechanisms and associations in these groups.

### Handling of overlapping datasets

We prioritized studies with the highest sample independence from the same research team using overlapping samples. For studies using the same sample to explore different dimensions (e.g., accelerometer vs. self-report data), we pooled effect sizes to prevent duplication. If multiple studies used the same dataset with highly overlapping goals (e.g., only adjusting the statistical model), we included the one with more comprehensive methodology or complete effect sizes.

### Resolution of discrepancies

After deduplication of the retrieved literature, two researchers independently screened the articles based on predefined inclusion and exclusion criteria. Initially, titles and abstracts were reviewed to identify potentially relevant studies for full-text examination. In addition to incorporating studies confirmed through full-text review, we also conducted a thorough examination of the reference lists of the retrieved full-text articles and other systematic reviews to ensure that no eligible studies were overlooked. The final selection of included articles was determined through joint verification by the two researchers. In cases of inconsistent screening results, a third researcher was consulted to make the final decision. To ensure consistency and reliability, we established a clear protocol for resolving discrepancies: initial disagreements were discussed between the two primary researchers, and if consensus could not be reached, the third researcher provided a final decision. This process ensured that all decisions were well-documented and transparent.

### Data extraction

Data were independently extracted by two authors based on the inclusion criteria, with discrepancies resolved through discussion. The following information was extracted:

(1) Author and publication year; (2) Study region; (3) Study design type; (4) Method of assessing neuroticism; (5) Method of assessing physical activity; (6) Statistical methods; (7) Association measures (correlation coefficients, standardized coefficients); (8) Study outcomes.

To ensure transparency and reproducibility, the data extraction form has been included as Table 1. This form details the specific variables and data points collected from each study, facilitating the replication of our data extraction process.

**Table 1:** Summary of the included literature.

**Number**	**Author, year**	**Study design**	**Country**	**Sample Size**	**Age (years) mean (SD) [range]**	**Percentage girls (%)**	**Instruments used (PA)**	**Instruments used (ES)**	**Analysis**	**Association indicators**	**Conclusion**
1	Sale et al., 2000	CS	UK	187	(24.69)	57.22%	Four-point Likert scale	EPI	Correlations	*r* = −0.10	The study results did not find a significant association between neuroticism and physical activity.
2	Saklofske et al., 2007	CS	CA	497	18–53 (24)	71.43%	EBQ	PMM	Correlations SEM	*r* = −0.14 γ = −0.45	Neuroticism is negatively associated with physical exercise behavior.
3	De Moor et al., 2008	LS	NL	7,558	18–50 (27.9)	64.14%	Four-point Likert scale	ABV	Correlations	*r* = −0.09	Lower levels of regular physical activity are associated with higher levels of anxiety and depressive symptoms.
4	Adams and Nettle, 2009	CS	US	423	18–50 (34.7)	81.82%	IPAQ	IPIP	Multiple regression	β = −0.18	There is no significant correlation between neuroticism and the frequency of physical activity.
5	Lochbaum et al., 2010	CS	US	1,484	(21)	55.72%	LTEQ	NEO	Correlations	*r* = −0.10 *r* = −0.06	Persistent engagement in long-term exercise may be associated with lower levels of neuroticism.
6	Brunes et al., 2013	CS	NED US UA NOR	38,743	>19 (51.2)	56.1%	IPAQ	EPQ	Multiple regression		Regular physical activity is significantly associated with lower neuroticism levels, with a stronger link in women. The frequency, duration, and intensity of activity all influence this negative correlation.
7	Bowen et al., 2013	LS	UK	3,374	>25	55.99%	Self-Report Measure	EPI-N	Multiple regression	β = −0.21	Individuals high in neuroticism tend to engage in physical exercise less frequently; however, maintaining a consistent exercise regimen can reduce emotional instability, thereby enhancing mental health.
8	Wilson et al., 2015	CS	US	409/298	18–20 (18.34)	100%	GPAQ, GLTEQ, IPAQ, NL-1000 piezoelectric accelerometer	IPIP	Correlations SEM	*r* = −0.269 γ= −0.608	Neuroticism is negatively correlated with objectively measured physical activity, whereas no significant association is observed with self-reported physical activity.
9	Wilson et al., 2016	CS	US	298	18–20 (18.34)	100%	GPAQ, GLTEQ, IPAQ	IPIP	Correlations	*r* = −0.112	When physical activity is objectively measured by an accelerometer, only individuals high in neuroticism and low in extraversion (neurotic-introverts) show a positive link between physical activity and better mental health. This suggests that physical activity's impact on mental health varies across personality types.
10	Sutin and Terracciano, 2016	CS	US	5,150	18–91 (44.61)	49.6%	Self-Report Measure	BFI	Multiple regression	β = −0.16	There is a significant negative correlation between neuroticism levels and physical activity. Individuals with higher neuroticism scores are more likely to reduce their daily physical activity.
11	Artese et al., 2017	CS	US	69	67–95 (80.2)	75.4%	ActiGraph ActiSleep	NEO	Multiple regression	β = −0.22	Elderly individuals with higher neuroticism scores engage in physical activity less frequently than their counterparts with lower neuroticism scores.
12	Chan et al., 2018	CS	HK UK	349	>50 (61.84)	55%	GSLTPAQ	BFI	Correlations	*r* = −0.11	Physical activity can be employed as an intervention strategy to help reduce the level of neuroticism in the elderly population.
13	Bessey, 2018	CS	US	110	18–20	44.55%	Self-Report Measure	BFI	Multiple regression	β = 0.0011	Under the experimental conditions of this study, neuroticism shows no significant link to physical activity, with a negligible effect size.
14	Kroencke et al., 2019	LS	US	3,243	18–24 (18.85)	60%	WSTS	BFI	Multiple regression	β = −0.119	There is a significant negative correlation between neuroticism and physical activity. This implies that individuals with lower levels of neuroticism are more likely to engage in physical activities with greater frequency.
15	Kekäläinen et al., 2020a	CS	FI	1,412	47–55 70–85	100% 59.9%	Actigraph accelerometer	EPI	Multiple regression	β = −0.081	Neuroticism in middle-aged women is associated with lower levels of leisure-time physical activity, whereas neuroticism is unrelated to physical activity in the elderly population.
16	Kekäläinen et al., 2020b	CS	FI	239	70–85 (74.71)	59%	Actigraph accelerometer	NEO	Multiple regression Correlations	β = −0.24 *r* = −0.12	Neuroticism overall shows no robust association with physical activity. However, impulsiveness, its only significant facet, is negatively correlated with accelerometer-assessed light activity.
17	Gacek et al., 2022	CS	POL ESP	499	18–35 (21.65)	37.88%	IPAQ	NEO	Multiple regression Correlations	β = −0.21 = −0.172	As levels of neuroticism increase, there is a significant decline in the levels of vigorous, moderate, and total physical activity, accompanied by an increase in sedentary time.
18	Satoh et al., 2022	CS	JP	1,141	28–65 (53.34)	100%	Self-Report Measure	TIPI-J	Correlations	OR = 0.390 *r* = −0.251	Among non-professional women, neuroticism is inversely associated with favorable physical activity habits.
19	Liao et al., 2022	LS	CN	9,284	12–15	48%	CEPS	CEPS	Multiple regression Correlations	β = 0.04 *r* = 0.07	Among Chinese adolescents, physical exercise positively predicts neuroticism, conscientiousness, and agreeableness through the mediating role of peer relationships. However, the positive link with neuroticism may be related to short-term emotion measurement and cultural context.
20	Thøgersen-Ntoumani et al., 2022	LS	DK/SE/NO/AU	297	(51)	72.4%	IPAQ-SF	API	Correlations	*r* = −0.23 *p* < 0.05 β = −0.16 *p* = 0.029	Neuroticism exerts a significant negative impact on physical activity. Interventions targeted at individuals with high levels of neuroticism, such as mindfulness and integrated programs, may facilitate their participation in physical activities.
21	Asquith et al., 2022	CS	UK	409	14–20 (16.62)	80%	Self-Report Measure	BFI	Correlations	*r* = −0.174 *p* < 0.05	Young individuals with high levels of neuroticism may need to enhance their wellbeing through appropriately increased physical activity. As individuals age, a decline in physical activity levels may exacerbate the negative impact of neuroticism on wellbeing.
22	Kekäläinen et al., 2023	CS	FI	239	70–85 (74.2)	59%	Actigraph accelerometer	NEO	Multiple regression, Correlations	β = −0.17	Older adults with low neuroticism are more likely to experience cognitive function improvements through physical training, indicating that low neuroticism may enhance physical activity's positive effects on cognition.
23	Desai et al., 2023	LS	US	7,685	(72)	62%	NHIS	NEO	Multiple regression	β = −0.17	Lower levels of neuroticism are associated with increased participation in physical activities; among females, higher levels of physical activity are correlated with lower neuroticism scores.
24	Campos-Uscanga et al., 2023	CS	MEX	579	18–59	38.86%	Self-Report Measure	BFI	Correlations	*r* = −0.207 *p* < 0.01 *r* = −0.141 *p* < 0.01	Neuroticism is negatively correlated with the total duration of physical activity, which may suggest that individuals with higher levels of neuroticism are likely to engage in physical activities for a shorter duration over the long term.
25	Ahola et al., 2024	LS	FI	141–307	33, 42, 50, 61	55%	Actigraph accelerometer	NEO	Correlations	*r* = −0.17 *p* < 0.05	Neuroticism is negatively associated with the total volume of physical activity, particularly moderate to vigorous physical activity.This suggests that individuals with higher levels of neuroticism are less likely to engage in physical activities, including those of moderate to high intensity.

CS, Cross-sectional study; LS, Longitudinal study; IPAQ, International Physical Activity Questionnaire; LETQ, Leisure-Time Exercise Questionnaire; GPAQ, Global Physical Activity Questionnaire; WSTS, Weekly self-tracking surveys; GSLTPAQ, Godin-Shephard Leisure-Time Physical Activity Questionnaire; EBQ, Exercise Behavior Questionnaire; BFI, Big Five Inventory; EPQ, Eysenck Personality Questionnaire; NEO, Revised NEO Personality Inventory; IPIP, International Personality Item Pool; TIPI-J, Japanese Version of the Ten Item Personality Inventory.

### Handling missing or ambiguous data

In cases where data were missing or ambiguous, we attempted to contact the corresponding authors of the original studies via email to request additional information or clarification. If the authors did not respond or if the data could not be obtained, we documented the missing data and conducted sensitivity analyses to assess the potential impact of these missing data on our results. Specifically, we performed both complete case analysis and multiple imputation methods to handle missing data, ensuring that our findings are robust and reliable.

### Literature quality assessment

The quality of the included studies was assessed using the QualSyst tool, which is designed for evaluating the methodological quality and bias in both quantitative and qualitative studies across different research designs (Kmet, 2004). The QualSyst tool consists of 14 items, but for this review, which includes observational studies, items 5 (random allocation), 6 (researcher blinding), and 7 (participant blinding) were omitted. Each item on the QualSyst tool is scored from 0 to 2, indicating whether the study meets a criterion (0 = no, 1 = partially, 2 = yes). The scores are summed to create a total score, which is then converted into a percentage by dividing by 22. The studies were rated as “excellent” (>80%), “good” (70%-79%), “adequate” (55%-69%), and “low” (< 55%) (Selzler et al., 2020; Fang et al., 2021). Two researchers independently assessed the quality, and any discrepancies were resolved through discussion until consensus was reached.

Although the QualSyst tool provides a comprehensive framework for evaluating methodological quality and bias, it may not fully capture certain biases, such as self-report bias, measurement error, or small sample bias. To address these limitations, we implemented additional checks and considerations. For studies relying solely on self-reported data, we critically evaluated the potential for bias and measurement error, noting whether validated instruments were used. These studies were scrutinized and rated more conservatively. Additionally, studies with small sample sizes (e.g., < 50 participants) were evaluated for potential sampling errors and external validity. While these studies met QualSyst criteria, we acknowledged their limitations and discussed these in the limitations section. To ensure the robustness of our findings, we conducted sensitivity analyses excluding studies with small sample sizes and those relying solely on self-reported data. The results of these analyses were consistent with our main findings, indicating that our conclusions are not unduly influenced by these potential biases.

### Statistical analysis

This study employed Stata 18 software for meta-analysis. The Spearman correlation coefficients (ρ) reported in the literature were converted to Pearson correlation coefficients (*r*) using the formula (*r* ≈ 6 sin(πρ/6)/π). Subsequently, the Pearson coefficients along with the sample sizes were transformed into Fisher's *Z*-scores, standard errors (SE), and 95% confidence intervals (95% CI). A random-effects model was used to conduct the meta-analysis of the correlation coefficients (*r*) and their 95% CI. The 95% CI for the standardized coefficients (β) was calculated using the formula (confidence interval = β ± 1.96 × SE).Based on the results of the heterogeneity test, either a fixed-effects model (I^2^ < 50%, *P* > 0.05) or a random-effects model (I^2^ ≥ 50% or *P* < 0.05) was employed for the analysis. The level of heterogeneity was assessed using the I^2^ index, categorized as low (I^2^ ≤ 25%), moderate (25% < I^2^ ≤ 50%), and high (I^2^ > 50%) (Liu et al., 2023; Higgins et al., 2003). In the presence of heterogeneity, subgroup analyses were conducted. Publication bias was assessed using funnel plots and Egger's test, and the results were illustrated with funnel plots.

Publication bias was evaluated using funnel plots and Egger's regression test. These methods were selected as standard approaches, though the authors acknowledge that additional techniques (e.g., Trim-and-Fill) could further contextualize findings.

## Results

### Study selection

**Figure 1:**
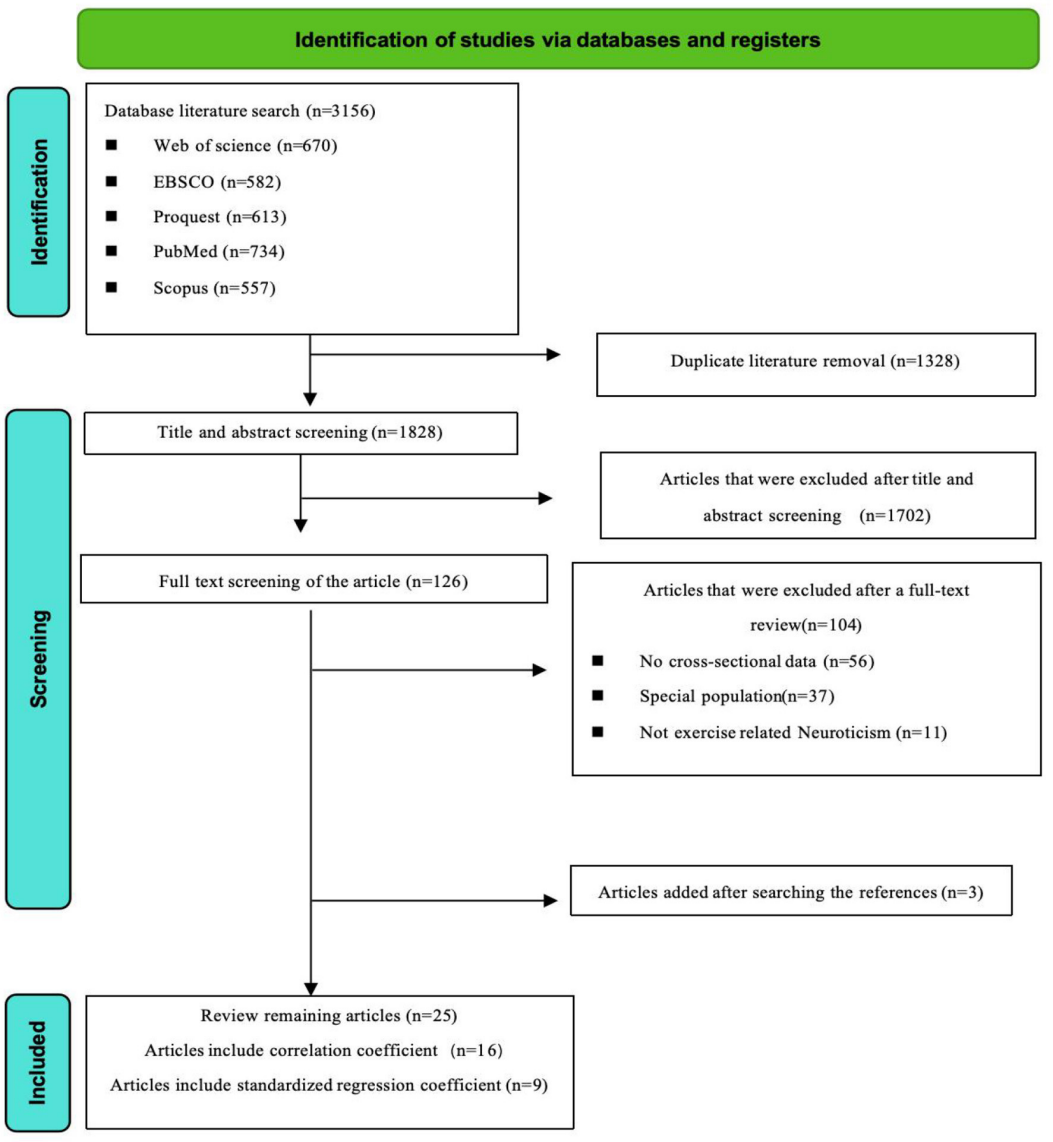
Literature screening process flowchart.

Figure 1 presents the screening process of the studies and the reasons for excluding articles. Initially, 3,156 potentially relevant articles were identified through database searches. After removing duplicates, 1,828 unique articles were subjected to title and abstract screening, resulting in the exclusion of 1,702 articles. The remaining 126 articles underwent full-text review, and ultimately, 104 articles were excluded for various reasons: 66 articles lacked original data, 27 studies focused on specific populations (such as those with diseases or athletes), and 11 studies did not involve exercise-related efficacy. Between January 2000 and October 2024, a total of 25 articles met the inclusion criteria.

### Basic literature information

Table 1 presents the basic characteristics of the 25 included studies. All were published post −2,000, consisting of 17 cross-sectional and eight longitudinal studies (with seven offering cross-sectional data). Sample sizes varied from 69 to 38,743 participants across countries like the US, UK, and Finland. In terms of analysis, 16 studies used correlation analysis to report *r-*values, and nine applied multiple regression analysis to provide β coefficients and SEs.

Notably, four studies (Kekäläinen et al., 2020a,b, 2023; Ahola et al., 2024) on the Finnish PASSWORD cohort, which explored neuroticism's links with accelerometer data, gait analysis, and long-term tracking results, were based on the same longitudinal cohort. To prevent sample duplication, complementary analysis dimensions (e.g., objective and subjective data) were combined. For the two studies by (Wilson et al. 2015), (2016) using the same sample of American female college students (*n* = 298), only objective physical activity outcomes were retained to avoid data duplication. The remaining studies span different age groups and regions, with specific details in Table 1.

### Quality scores and interpretation

The quality assessment of the 25 included studies, utilizing the QualSyst tool, yielded scores ranging from 72.7% to 100%, with a mean score of 89.84%. The majority of studies (24, 96%) were categorized as high quality, reflecting robust adherence to methodological standards, while one study (4%) was rated as good quality. These scores underscore the overall high methodological rigor of the included studies. Detailed quality scores for each study are provided in Table 2. A high-quality score signifies that a study has met the majority of the methodological criteria, whereas a lower score indicates potential methodological limitations that may influence the study's findings.

**Table 2:** Results of the quality assessment of the studies included in the systematic review (*N* = 25).

**Author and year**	**1. Research question**	**2. Study design**	**3. Subject and variable selection**	**4. Subject characteristics**	**8. Exposures and outcome**	**9. Sample size**	**10. Analytic methods**	**11. Estimate of variance**	**12. Confounding**	**13. Results in sufficient detail**	**14. Conclusions supporting results**	**Sums**	**Weights ^†^**	**Rank ^#^**
Sale et al., 2000	2	2	2	2	1	1	2	2	2	2	2	20	90.9%	Excellent
Saklofske et al., 2007	2	2	1	2	2	2	2	2	2	2	2	21	95.5%	Excellent
De Moor et al., 2008	2	2	2	2	1	2	2	2	2	2	2	21	95.5%	Excellent
Adams and Nettle, 2009	2	1	2	2	1	1	2	2	2	2	2	19	86.4%	Excellent
Lochbaum et al., 2010	2	1	2	2	2	2	2	1	1	2	2	19	86.4%	Excellent
Brunes et al., 2013	2	2	2	2	2	2	2	1	2	2	2	21	95.5%	Excellent
Bowen et al., 2013	2	2	2	2	2	2	2	2	2	2	2	22	100.0%	Excellent
Wilson et al., 2015	2	2	2	2	2	1	2	1	1	2	2	19	86.4%	Excellent
Wilson et al., 2016	2	2	2	2	2	2	2	1	2	2	2	21	95.5%	Excellent
Sutin et al., 2016	2	2	2	2	2	2	2	1	2	2	2	21	95.5%	Excellent
Artese et al., 2017	2	2	1	2	2	0	2	1	2	2	2	18	81.8%	Excellent
Chan et al., 2018	2	1	1	2	2	1	2	2	1	2	2	18	81.8%	Excellent
Bessey, 2018	2	2	1	2	2	0	2	2	1	2	2	18	81.8%	Excellent
Kroencke et al., 2019	2	2	1	2	2	1	2	2	1	2	2	19	86.4%	Excellent
Kekäläinen et al., 2020a	2	2	2	2	2	1	2	1	1	2	2	19	86.4%	Excellent
Kekäläinen et al., 2020b	2	2	1	2	2	1	2	2	1	2	2	19	86.4%	Excellent
Gacek et al., 2022	2	2	1	1	1	1	2	2	0	2	2	16	72.7%	Good
Satoh et al., 2022	2	2	2	2	1	1	2	1	1	2	2	18	81.8%	Excellent
Liao et al., 2022	2	2	2	2	1	2	2	2	2	2	2	21	95.5%	Excellent
Thøgersen-Ntoumani et al., 2022	2	2	2	2	2	2	2	1	2	2	2	21	95.5%	Excellent
Asquith et al., 2022	2	2	1	2	2	1	2	2	1	2	2	19	86.4%	Excellent
Kekäläinen et al., 2023	2	2	2	2	2	2	2	2	2	2	2	22	100.0%	Excellent
Desai et al., 2023	2	2	2	2	2	2	2	1	2	2	2	21	95.5%	Excellent
Campos-Uscanga et al., 2023	2	2	2	2	2	2	2	2	1	2	2	21	95.5%	Excellent
Ahola et al., 2024	2	2	2	2	2	1	2	1	2	2	2	20	90.9%	Excellent

Each item was scored depending on to what degree the criterion was met: yes = 2 points, partial = 1 point, no = 0.

^†^Study total sum score divided by the total possible score of 22.

^#^A percentage score of >80%, 70–80%, 55–69% and < 55% was rated as “excellent,” “good,” “adequate,” and “low”, respectively.

Most included studies scored highly on the QualSyst assessment tool. However, this tool may be insensitive to methodological limitations, particularly for studies with small sample sizes or those relying solely on self-reported data. Studies with small samples (e.g., < 500) are prone to sampling-related errors, which can restrict external validity. Meanwhile, studies using only self-reported data may be subject to measurement subjectivity, introducing potential bias. These issues might not be fully captured in the quality scores. As shown in Table 2, some highly-rated studies (e.g., Sale et al., 2000; Saklofske et al., 2007), despite meeting methodological rigor standards, have small sample sizes or depend on self-reported data. These factors can undermine the robustness and generalizability of their results.

### Handling of low-quality studies

While the majority of the included studies were rated as high quality, we acknowledge that some studies, particularly those relying solely on self-reported data or with small sample sizes, may introduce bias. To address this, we conducted sensitivity analyses by excluding studies that used self-reported measurement tools and those with sample sizes fewer than 50 participants. The results of these sensitivity analyses were consistent with our main findings, indicating that our conclusions are robust and not unduly influenced by these potential biases.

### Assessment tools for physical activity

In the literature included in this study, a total of 11 tools were employed to assess the frequency and energy expenditure of physical activity. Among them, nine studies utilized self-reported methods to evaluate physical activity by inquiring about the frequency, duration, and intensity of participants' activities within specific time frames. This approach aids researchers in understanding the overall activity level of subjects (Likert, 1932). Six articles employed the International Physical Activity Questionnaire (IPAQ) (Craig et al., 2003), which calculates an individual's weekly physical activity volume and distinguishes between different intensities of physical activity. Four studies utilized the Actigraph accelerometer as an assessment tool, wearing monitors to collect objective physical activity data (Hendelman et al., 2000). Two articles used the Global Physical Activity Questionnaire (GPAQ) to monitor trends in physical activity and assess the impact of physical activity on health (Armstrong and Bull, 2006). The Godin Leisure-Time Exercise Questionnaire (GLTEQ) was employed in two studies, aiming to evaluate the level of physical activity during leisure time (Sikes et al., 2019). One study used a Exercise Behavior Questionnaire to gather information on the frequency, intensity, and duration of individual exercise routines, assessing exercise habits and participation (Saklofske et al., 2007). Additionally, one study also utilized the ActiGraph ActiSleep monitor to collect objective, detailed, and reliable physical activity data (Ancoli-Israel et al., 2003). The Leisure Time Exercise Questionnaire (LTEQ) was used in one article to assess participants' exercise behaviors by inquiring about the frequency of light, moderate, and vigorous exercise per week (Jacobs et al., 1993). One study used the Godin Leisure-Time Physical Activity Questionnaire (GSLTPAQ), which specifically targets physical activity during leisure time, excluding activities related to work or daily routines (Godin and Shephard, 1985). One article used the Weekly Self-Tracking Questionnaire (WSTQ) to collect data on exercise frequency (number of exercise days in the past week, ranging from 0 to 7 days), assisting health professionals in assessing physical activity levels and providing (Kroencke et al., 2019) personalized advice and interventions. One study calculated exercise time through the “exercise duration from Monday to Friday” and “exercise duration on weekends” measures in the China Education Panel Survey (CEPS). These assessment tools offer a multifaceted evaluation of physical activity, covering activity frequency and the quantitative analysis of energy expenditure using MET values (Liao et al., 2022).

### Assessment tools for neuroticism

In the included literature, ten primary tools were employed to assess the neuroticism level associated with individual participation in physical activities. Six studies utilized the Big Five Personality Inventory (BFI), which is one of the instruments for evaluating the Big Five personality traits and is used to assess the propensity for emotional stability, with higher scores potentially indicating a greater likelihood of experiencing anxiety, depression, and other negative emotions (McCrae and Costa, 1987). Four articles employed the Eysenck Personality Questionnaire (EPQ), in which the Neuroticism scale (N) is a subscale used to assess neuroticism (Eysenck and Eysenck, 1985). Three studies used the Eysenck Personality Inventory (EPI), which measures an individual's emotional stability and tendency toward emotional reactions through a series of self-reported questionnaire items (McCrae and John, 1992). One study used the Personality Mini-Markers (PMM), which typically assesses emotional stability and the ability to cope with stress through reverse scoring for neuroticism (Saucier, 1994). Additionally, three studies utilized the International Personality Item Pool (IPIP), whose neuroticism scale is not only useful for studying personality traits but also for assessing the risk of mental health issues and aiding in diagnostic and treatment planning in clinical settings (Goldberg et al., 2006). Seven studies used the NEO Personality Inventory (NEO-PI), in which the neuroticism dimension is defined as the opposite of emotional stability, with higher scores often indicating greater emotional volatility, while lower scores suggest relative stability and calmness (Costa and McCrae, 2000). One study used the Amsterdamse Biografische Vragenlijst (ABV), where the neuroticism dimension involves assessing the frequency and intensity of negative emotions experienced by individuals, such as anxiety, depression, anger, self-consciousness, and vulnerability (De Moor et al., 2008). One study used the Australian Personality Inventory (API), with higher neuroticism scores typically indicating a greater tendency to experience negative emotions like anxiety, depression, and self-doubt, while lower scores suggest relative emotional stability (Murray et al., 2009). One article measured neuroticism through five items in the China Education Panel Survey (CEPS) (Guo et al., 2020). One article used the Ten-Item Personality Inventory (TIPI-J), which assesses the neuroticism personality trait through two specific items; in the TIPI-J, neuroticism is commonly associated with emotional instability, anxiety, and irritability (Oshio et al., 2013).

### Correlation between physical activity and neuroticism

**Figure 2:**
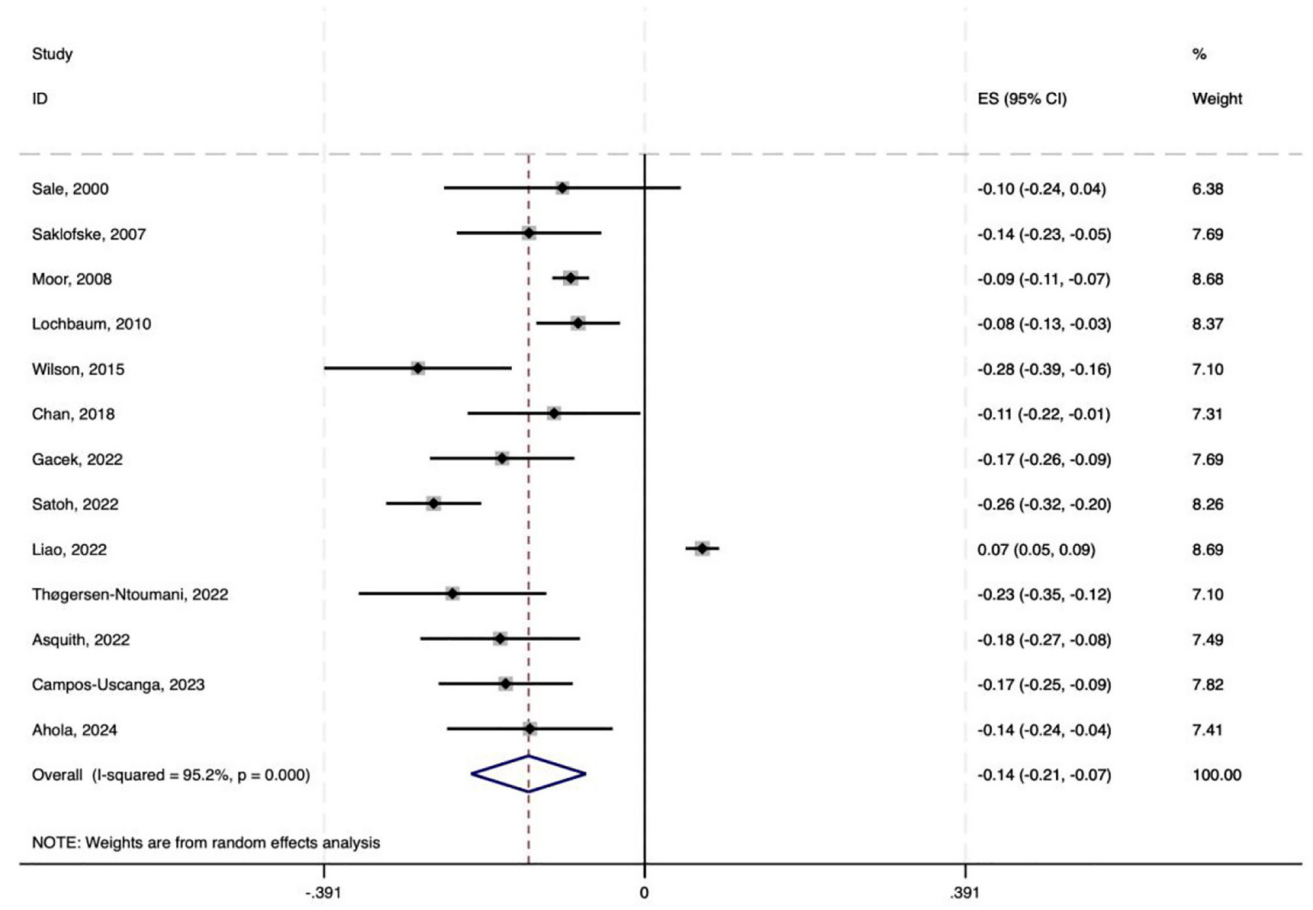
Forest plot of correlation coefficients.

Among the 25 included studies, 15 provided data on the correlation between physical activity and Neuroticism. To standardize the analysis, Spearman's rho (ρ) coefficients reported in the literature were converted to Pearson's *r* coefficients via the following formula: *r* ≈ 6 sin(πρ/6)/π. These coefficients were then transformed into Fisher's *Z-*scores for meta-analysis, along with the calculation of standard errors (SE) and 95% confidence intervals (CI) (Borenstein et al., 2011; Szczuka et al., 2021). To address excessive representation of single-team studies and repeated samples (e.g., those by Kekäläinen and Wilson), we aggregated their effect sizes. The results showed a combined effect size of −0.142, with a 95% confidence interval ranging from −0.212 to −0.072, indicating a significant effect across studies. The heterogeneity test results revealed a chi-square value of 251.63 with 12 degrees of freedom, and a *p*-value < 0.0001, indicating significant heterogeneity among studies. The *I*-squared value was 95.2%, suggesting that 95.2% of the variation in effect size can be attributed to heterogeneity between studies. Additionally, the *z*-value for the Test of ES = 0 was 3.96, with a *p*-value < 0.0001, further confirming the statistical significance of the results (Figure 2).

Converting the pooled Fisher's *Z-*scores and 95% CIs back to Pearson's *r*, the average correlation coefficient was found to be −0.141, with a 95% CI ranging from approximately −0.209 to −0.072. Therefore, the average correlation between physical activity and is neuroticism −0.141, with a 95% CI of −0.209 to −0.072.

**Figure 3:**
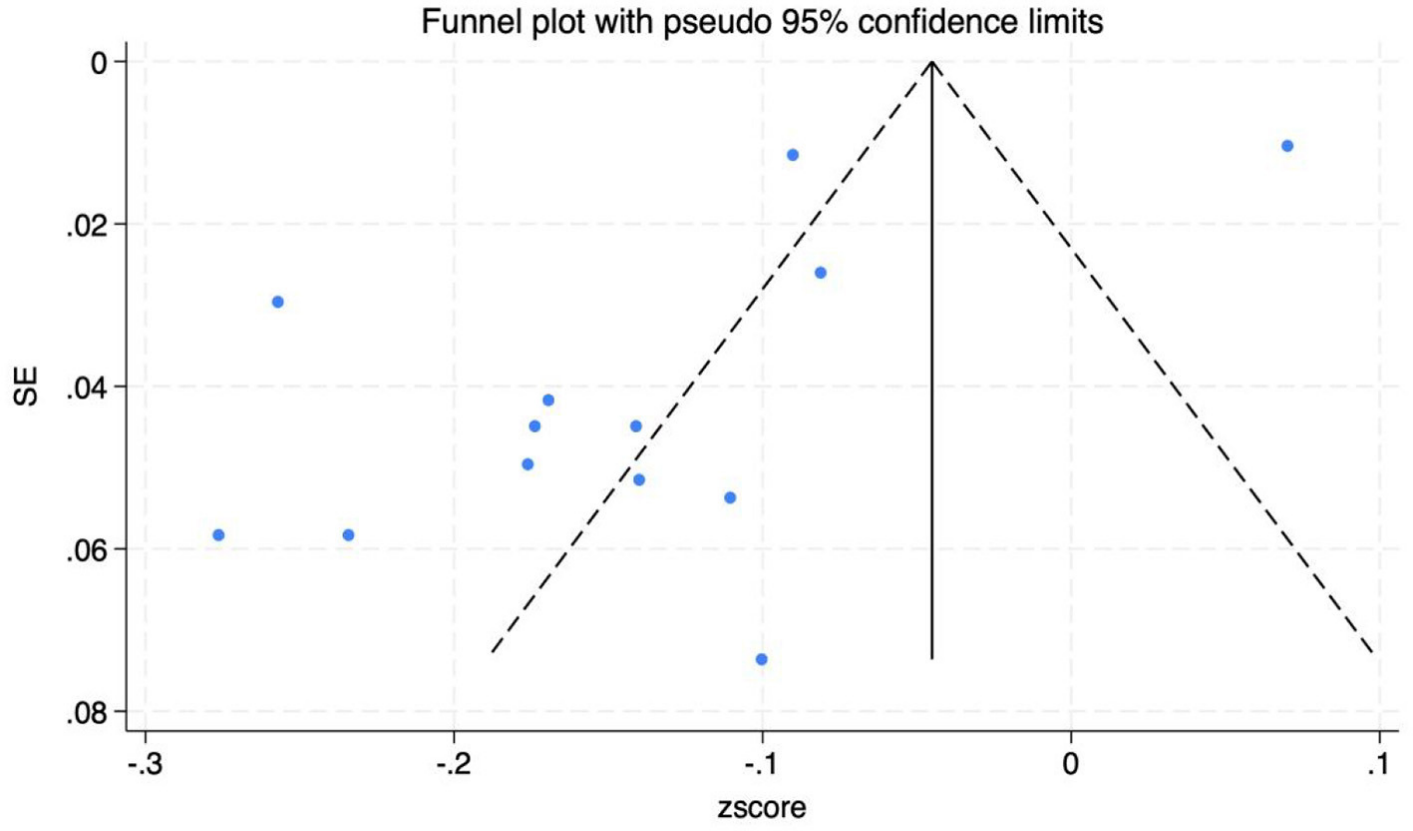
Funnel plot of correlation analysis.

Funnel plots and Egger's test were utilized to assess publication bias among the included studies. The symmetry of the funnel plot (Figure 3) and the *P*-value of the intercept coefficient from Egger's test (0.294) indicate that there is no publication bias in the statistical correlation data *r*.

### Impact of neuroticism on physical activity

Among the 25 studies included, 12 employed multiple regression analysis to provide effect sizes (standardized coefficients β) of neuroticism on physical activity. The 95% confidence intervals were calculated via the following formula: 95% CI = β ± 1.96 × SE. A random-effects model was used to conduct the meta-analysis on the standardized coefficients β and their 95% CIs. To address over-inclusion of single-team studies and duplicate samples (e.g., in Kekäläinen et al.'s study), we aggregated their effect sizes.

**Figure 4:**
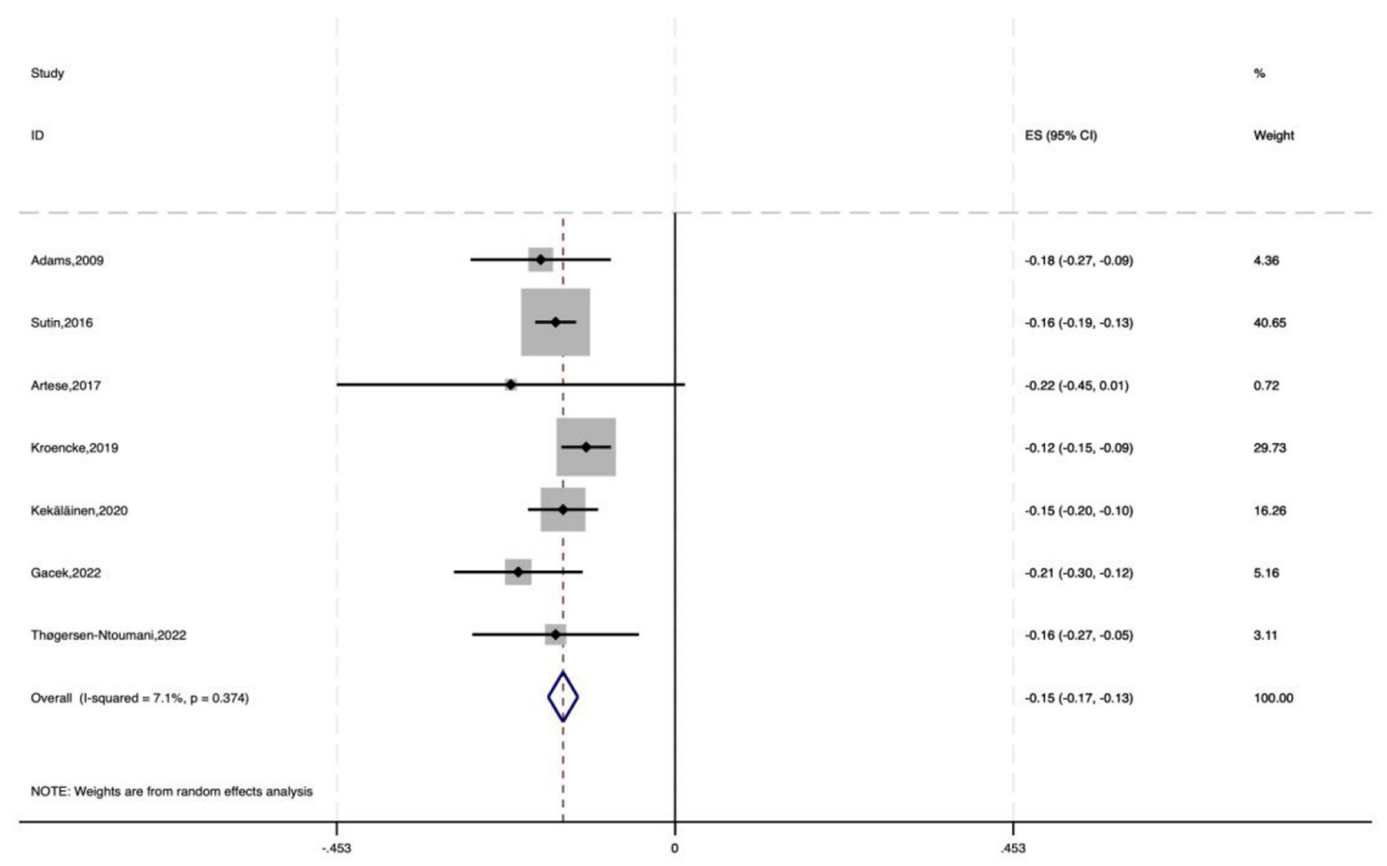
Forest plot of the impact of neuroticism on physical activity (random-effects meta-analysis).

The analysis showed a pooled effect size of −0.150 (95% CI: −0.170 to −0.130), indicating a significant negative impact of neuroticism on physical activity. Heterogeneity testing (χ^2^ = 6.46, df = 6, *P* = 0.374) revealed no significant heterogeneity between studies. The *I*^2^ value of 7.1% shows that only 7.1% of effect size variability was due to between—study heterogeneity. Moreover, the *z*-value for testing ES = 0 was 14.79 (*P* < 0.001), further confirming the statistical significance (Figure 4).

Overall, these results indicate that neuroticism has a negative effect on physical activity, with a standardized coefficient of −0.150. This means that for each 1 – SD increase in neuroticism, physical activity decreases by an average of 0.150 SD units.

**Figure 5:**
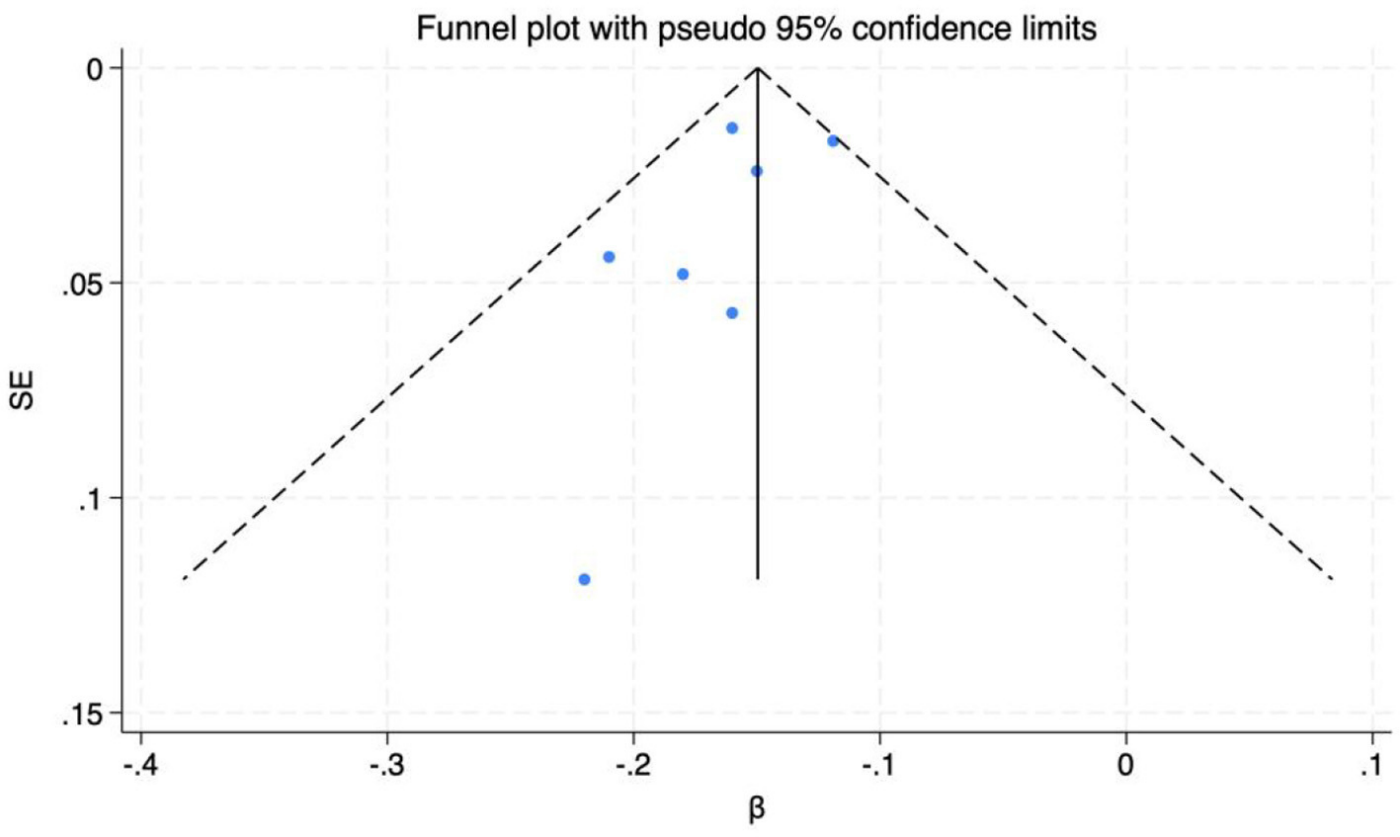
Funnel plot assessing publication bias for the impact of neuroticism on physical activity.

Funnel plots and Egger's test were employed to assess publication bias among the included studies. The symmetry of the funnel plot (Figure 5) and the *P*-value of the intercept coefficient from Egger's test (0.272) indicate that there is no publication bias in the statistical analysis of the standardized coefficient β.

### Impact of physical activity on neuroticism

Of the 25 studies included, 12 used multiple regression analysis, and 3 of these provided effect sizes (standardized coefficients β) for the impact of physical activity on neuroticism. Based on the standardized coefficient β and standard error (SE), the 95% confidence interval (CI) was calculated using the formula (CI = β ± 1.96 × SE). A random-effects model was used for the meta-analysis of β and the 95% CI.

**Figure 6:**
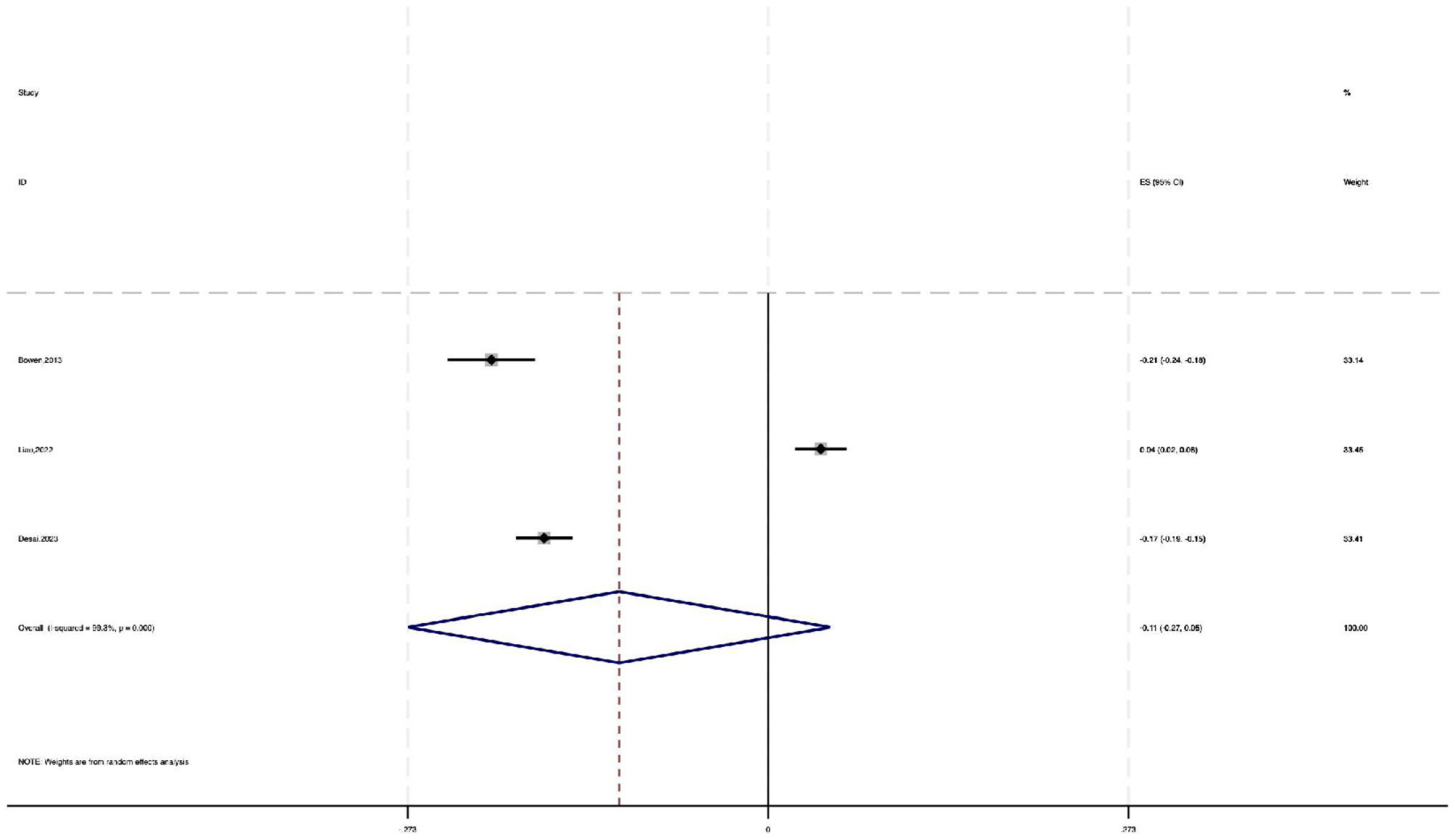
Forest plot of the effect of physical activity on neuroticism (random-effects meta-analysis).

The meta-analysis showed a pooled effect size of −0.113 (95% CI: −0.273 to 0.047). As the CI includes zero, there's no significant overall effect of physical activity on neuroticism across the 3 studies. Heterogeneity testing (χ^2^ = 269.42, df = 2, *P* < 0.0001) indicated significant statistical heterogeneity between studies. The *I*^2^ value of 99.3% suggests that 99.3% of effect size variability is due to between—study heterogeneity. Also, the *z*-value for testing ES = 0 was 1.38 (*P* = 0.167), indicating statistical nonsignificance (Figure 6). Overall, the results show no significant effect of physical activity on neuroticism (pooled standardized coefficient = −0.113). Although a slight negative correlation exists, it may not be practically significant due to high heterogeneity and nonsignificant effect sizes. This might be due to the small number of included studies and measurement tool errors.

**Figure 7:**
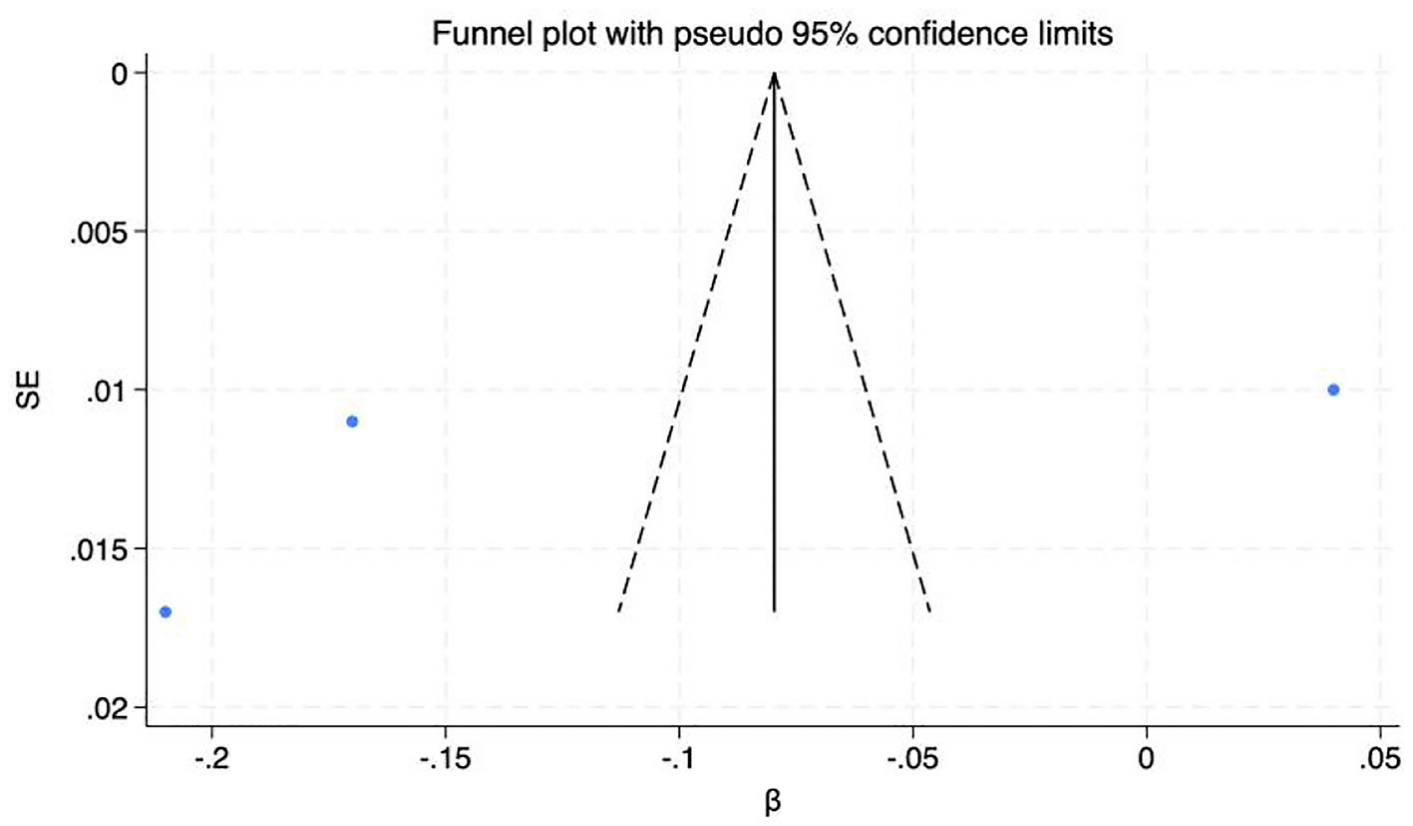
Funnel plot assessing publication bias for the effect of physical activity on neuroticism.

A funnel plot and Egger's test were employed to assess publication bias among the included studies. The symmetry of the funnel plot (Figure 7) and the *P*-value of the intercept coefficient from Egger's test (0.540) do not provide sufficient evidence to indicate statistically significant publication bias in the standardized coefficient β. However, further validation is required by considering the number of studies, the context of the field, and other bias assessment methods.

Egger's test and funnel plots indicated no evidence of publication bias; therefore, the trim-and-fill method was not applied.

### Subgroup analyses

To elucidate sources of heterogeneity, we conducted stratified analyses by age and by assessment modality. Age cut-offs (< 25, 25–60, > 60 yr) were selected to align with established developmental transitions—emerging adulthood, mid-life, and older adulthood—commonly employed in Five-Factor Model research (Sutin and Terracciano, 2016; Kekäläinen et al., 2023) and the WHO Global Physical Activity Surveillance framework.

Despite this stratification, substantial residual heterogeneity persisted across age subgroups (*I*^2^ = 88–95 %) and tool subgroups (*I*^2^ = 48–98 %). Consequently, pooled estimates should be interpreted cautiously; future individual-level or meta-regression analyses are warranted to clarify the influence of unmeasured moderators such as culture, comorbidity, or seasonality.

Moreover, only three studies employed accelerometers for objective measurement, limiting the precision and generalisability of the accelerometer-specific estimate (*r* = −0.18, 95 % CI −0.27 to −0.09). Replication with larger, objectively monitored cohorts is essential before drawing definitive conclusions.

### Age subgroups

To explore how different age groups might influence the results, we conducted a subgroup analysis. The data was divided into three subgroups: Under 25 years old, From 25 to 60 years old, and Above 60 years of age. The Under 25 years old subgroup included seven studies; the From 25 to 60 years old subgroup had five studies; and the Above 60 years of age subgroup contained two studies. For each age subgroup, we deeply analyzed the correlation between physical activity and neuroticism. The results of the age-based subgroup analysis showed that, in all age groups, there was a significant negative correlation between physical activity and neuroticism.

**Figure 8:**
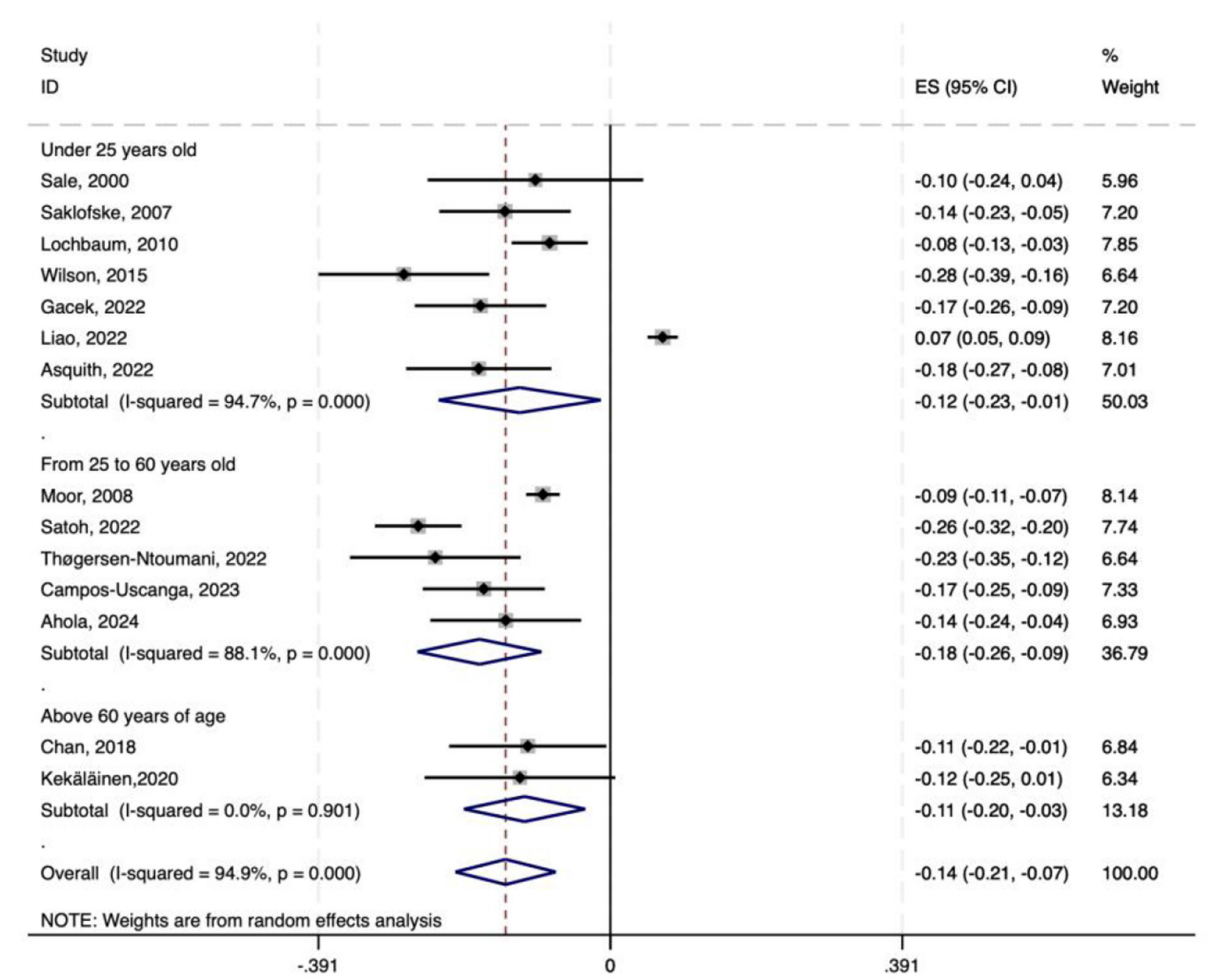
Forest plot of age-based subgroup analysis on the correlation between physical activity and neuroticism.

The subgroup analysis revealed that the pooled effect size for the under 25 years old group was −0.121 (95% CI: −0.230 to −0.013), that for the 25 to 60 years old group was −0.175 (95% CI: −0.258 to −0.092), and that for the above 60 years of age group was −0.115 (95% CI: −0.196 to −0.034). The *p*-values for all three subgroups were < 0.001, indicating a significant effect across studies. However, high heterogeneity was observed in the under 25 years old group (*I*^2^ = 94.7%) and the From 25 to 60 years old group (*I*^2^ = 88.1%). This might be due to differences among studies in design, choice of measurement tools, and sample characteristics. In contrast, the Above 60 years of age group had low heterogeneity (*I*^2^ = 0.0%), with highly consistent results. But with only two studies and a small sample size, its conclusions need to be verified by further studies with larger sample sizes to confirm stability and generalizability (Figure 8).

The combined Fisher's *z-*scores and 95% CIs were transformed back to correlation coefficients *r* and their 95% CIs. The correlation coefficient *r* between physical activity and neuroticism was −0.120 (95% CI: −0.226 to −0.013) in the under 25 age group, −0.173 (95% CI: −0.225 to −0.092) in the 25 to 60 age group, and −0.114 (95% CI: −0.193 to −0.034) in the over 60 age group. Thus, the average correlation coefficients in the under 25, 25–60, and over 60 groups were −0.120, −0.173, and −0.114 respectively. All confidence intervals excluded zero (*p* < 0.001), indicating statistically significant negative correlations across age subgroups.

### Tool subgroups

To explore the impact of different physical activity assessment tools on the results, we performed a subgroup analysis based on tool type. The data were divided into three subgroups: standardized self-report questionnaires (5 studies), accelerometers (3 studies), and other self-report measures (6 studies).

**Figure 9:**
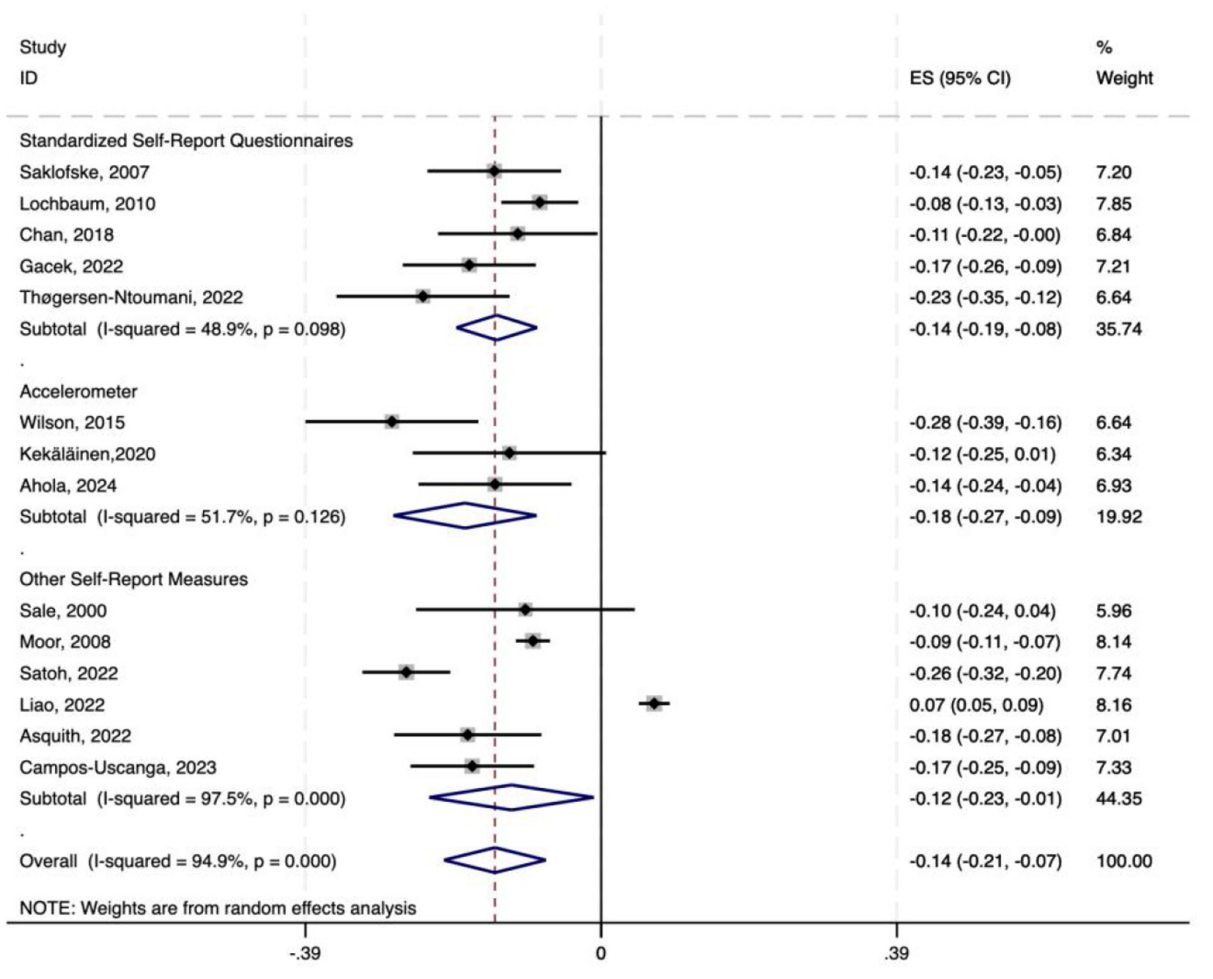
Forest plot of subgroup analysis on the correlation between physical activity and neuroticism based on different physical activity assessment tools.

In all subgroups, a significant negative correlation between physical activity and neuroticism was found. The subgroup using standardized self-report questionnaires showed a pooled effect size of −0.138 (95% CI: −0.191 to −0.084), the accelerometer subgroup had a pooled effect size of −0.180 (95% CI: −0.274 to −0.085), and the subgroup using other self-report measures had a pooled effect size of −0.118 (95% CI: −0.227 to −0.010). All subgroups had *P-*values below 0.001. Heterogeneity testing revealed moderate heterogeneity in the standardized self-report questionnaire subgroup (*I*^2^ = 48.9%) and accelerometer subgroup (*I*^2^ = 51.7%), likely due to tool design differences or inconsistent sample characteristics. The other self-report measures subgroup exhibited high heterogeneity (*I*^2^ = 97.5%), probably caused by measurement errors from non-standardized tools or vague concept definitions (see Figure 9).

Given the small sample size of the accelerometer subgroup (*n* = 3), its conclusions should be interpreted cautiously. Future research should prioritize standardized tools to reduce heterogeneity and increase the accelerometer-based study sample size to enhance result reliability and comparability.

The combined Fisher's *z-*scores and 95% CIs were transformed back to correlation coefficients *r* and their 95% CIs. Across all subgroups, significant negative correlations between physical activity and neuroticism were observed (*p* < 0.001). Specifically, the standardized self-report questionnaire subgroup showed a correlation coefficient of *r* = −0.137 (95% CI: −0.189, −0.084), the accelerometer-based measurement subgroup had *r* = −0.178 (95% CI: −0.267, −0.085), and the other self-report measurement subgroup demonstrated *r* = −0.118 (95% CI: −0.223, −0.010). All confidence intervals excluded zero, confirming statistically robust negative associations. The strongest correlation was found in the accelerometer subgroup (*r* = −0.178), while the weakest magnitude was seen in the other self-report subgroup (*r* = −0.118). These results highlight consistent age- and measurement-specific links between reduced physical activity and higher neuroticism.

In our assessment tool-based subgroup analysis of the correlation between physical activity and neuroticism, we found that the choice of measurement tool significantly impacts the observed correlation. Specifically, the subgroup using standardized self-report questionnaires showed a correlation coefficient of −0.138, the accelerometer-based subgroup demonstrated a stronger correlation at −0.180, and the subgroup using other self-report measures had a coefficient of −0.118. All subgroups showed a significant negative correlation (*p* < 0.05). The larger effect size in the accelerometer subgroup suggests that objective measurement tools might more accurately capture the relationship between physical activity and neuroticism than subjective self-report tools. However, significant heterogeneity between subgroups (*p* < 0.05) indicates that factors beyond tool type also influence this relationship. To better understand the specific mechanisms at play, future research should control for additional potential confounding variables.

### Effect size synthesis

The meta-analysis of correlation coefficients unveiled a significant negative correlation between physical activity and neuroticism (average correlation coefficient *r* = −0.141, *p* < 0.001). This suggests that individuals with higher levels of neuroticism are inclined to engage in less physical activity. Specifically, for every one standard deviation increase in neuroticism, physical activity levels decrease by ~0.141 standard deviation units. Although this correlation is statistically significant, its practical significance should be interpreted with caution. In real-world applications, such as mental health interventions, reducing neuroticism levels may only have a modest impact on increasing physical activity levels. This implies that additional factors, such as social support and environmental factors, should be considered to enhance the effectiveness of interventions.

The meta-analysis of multiple regression analyses further demonstrated that neuroticism significantly inhibits physical activity engagement (average standardized coefficient β = −0.15, *p* < 0.001). This indicates that for each one standard deviation increase in neuroticism, physical activity levels decrease by an average of 0.15 standard deviation units. This finding underscores the substantial inhibitory effect of neuroticism on physical activity.

However, significant heterogeneity was observed across studies (*I*^2^ > 90% ), indicating substantial variability in effect sizes. To better understand the sources of this heterogeneity, future research should conduct meta-regression analyses to systematically explore potential moderators, such as age, gender, cultural background, measurement tools, and study design. Additionally, subgroup analyses based on gender could provide further insights into the differential impact of neuroticism on physical activity among males and females.

Given the predominance of cross-sectional studies in our meta-analysis, it is important to note that the observed negative correlation between neuroticism and physical activity may reflect a bidirectional relationship. While higher neuroticism levels may inhibit physical activity participation, lower physical activity levels could also exacerbate neuroticism. Therefore, causal inferences cannot be drawn solely from cross-sectional data. Future research should prioritize longitudinal and experimental designs to establish the directionality and causality of the relationship between neuroticism and physical activity. For instance, intervention studies that aim to reduce neuroticism levels could assess the long-term impact on physical activity engagement, and vice versa, to provide more robust evidence for developing targeted interventions.

### Sensitivity analyses

To assess the robustness of our findings, we conducted sensitivity analyses by excluding studies that relied solely on self-reported measures of physical activity. The remaining studies included objective measures of physical activity, such as accelerometers. The sensitivity analysis included three studies: Wilson and Dishman (2015), Kekäläinen et al. (2020b), and Ahola et al. (2024).

The average correlation coefficient for the remaining studies was *r* = −0.160 (95% CI: −0.270 to −0.050). This indicates a significant negative association between neuroticism and physical activity, with a slightly larger effect size compared to the full sample analysis (original analysis: *r* = −0.141, 95% CI: −0.212 to −0.072).

These results suggest that studies using objective measures of physical activity tend to report a stronger negative association between neuroticism and physical activity compared to those relying on self-reported data. This highlights the importance of using objective measurement tools in future research to provide more reliable estimates of the relationship between neuroticism and physical activity.

### Factors influencing the correlation between physical activity and neuroticism

There are external influencing factors between neuroticism and physical activity, including individual differences (17 studies, 68%) (Sale et al., 2000; Saklofske et al., 2007; De Moor et al., 2008; Lochbaum et al., 2010; Brunes et al., 2013; Wilson et al., 2015, 2016; Sutin and Terracciano, 2016; Artese et al., 2017; Kroencke et al., 2019; Gacek et al., 2022; Satoh et al., 2022; Liao et al., 2022; Thøgersen-Ntoumani et al., 2022; Kekäläinen et al., 2023; Desai et al., 2023; Ahola et al., 2024), type of physical activity (nine studies, 36%) (Saklofske et al., 2007; Lochbaum et al., 2010; Brunes et al., 2013; Artese et al., 2017; Gacek et al., 2022; Liao et al., 2022; Kekäläinen et al., 2023; Desai et al., 2023; Campos-Uscanga et al., 2023), gender (six studies, 24%) (Sale et al., 2000; Saklofske et al., 2007; Brunes et al., 2013; Sutin and Terracciano, 2016; Kroencke et al., 2019; Campos-Uscanga et al., 2023), age (five studies, 20%) (Saklofske et al., 2007; Brunes et al., 2013; Sutin and Terracciano, 2016; Kroencke et al., 2019; Asquith et al., 2022), and social support (four studies, 16%) (Saklofske et al., 2007; Adams and Nettle, 2009; Lochbaum et al., 2010; Asquith et al., 2022). These factors collectively influence the relationship between physical activity and neuroticism.

### Quantitative evidence on external moderators

Few studies provided quantitative data on external moderators. Brunes et al. (2013) reported a stronger neuroticism–physical activity correlation in females (*r* = −0.26) than in males (*r* = −0.14), suggesting a potential moderating role of gender. Saklofske et al. (2007) noted that the effect of neuroticism on physical activity was attenuated under high social support (β = −0.21) compared to low support (β = −0.45), indicating that social support might mitigate the impact of neuroticism on physical activity. These findings imply that gender and social support could be important moderators in the relationship between neuroticism and physical activity. However, formal moderator analyses were impeded by the limited number of studies providing such data and the inconsistent reporting formats. Future research should employ consistent measurement tools and reporting formats to facilitate formal meta-regression or subgroup analyses of these potential moderators.

## Discussion

This study is the first to comprehensively and systematically review the association between physical activity and neuroticism. Through systematic database searches, we identified 25 research articles, encompassing both cross-sectional and longitudinal study designs, with an overall high quality of literature. The research methods primarily involved correlation analysis and multiple regression analysis. Standardized processing was applied to similar correlation data, followed by meta-analysis. The results indicate a significant statistical association between physical activity and neuroticism.

To further explore this correlation, we performed subgroup analyses on the correlation data between physical activity and neuroticism, categorizing studies by three age groups: under 25, 25–60, and over 60. Significant negative correlations emerged in all subgroups, indicating that physical activity impacts neuroticism across age groups, showing a certain universality. However, significant heterogeneity between subgroups was found, likely due to varying sample age ranges. For instance, Brunes et al. (2013) noted that physical activity levels decline with age, while neuroticism levels may rise. This aligns with McDowell et al. (2020), suggesting that personality traits can change with age, making the relationship between neuroticism and physical activity less pronounced in older adults.

Additionally, to further explore the correlation between physical activity and neuroticism, we performed subgroup analyses based on different physical activity assessment tools. These analyses divided the data into three subgroups: standardized self-report questionnaires, accelerometers, and other self-report tools. The analysis showed a significant negative correlation between physical activity and neuroticism across all subgroups. This indicates that the effect of physical activity on neuroticism is significant and universal, regardless of the assessment tool used. However, significant heterogeneity was found among the subgroups, which may be due to differences in tool design and sample characteristics.

When analyzing heterogeneity, we used subgroup analyses to examine the impacts of age groups and physical activity assessment tools. But to more comprehensively explore its sources, we found that personality and sample types or study regions might also be key contributors. For instance, different personality assessment tools can vary in their measurement of neuroticism. Clinical and general populations may differ in neuroticism and physical activity levels. Regional differences in culture, lifestyle, and physical activity facilities can also affect results. Due to data limitations, we couldn't perform meta-regression analyses or more in-depth sensitivity analyses. We acknowledge these limitations might influence our findings and suggest future research consider these factors in design and data analysis for better result interpretation.

To enhance the reliability of the study results, we further conducted publication bias tests on the included literature. Using funnel plots and Egger's test, we found no evidence of publication bias in the correlation data *r* and the standardized coefficients β. These findings provide an important empirical basis for further exploring the role of neuroticism in promoting individual physical activity and offer references for future research directions and methodologies.

### The association mechanism between physical activity and neuroticism

The results of the meta-analysis of correlation coefficients revealed a moderate negative correlation between physical activity and neuroticism (average correlation coefficient *r* = −0.141, *p* < 0.001), indicating that individuals who actively engage in physical activity tend to have lower levels of neurotic personality traits. This finding is consistent with the conclusions of Allen and Laborde (2014), suggesting that lower neuroticism is associated with higher levels of physical activity. However, Adams and Nettle (2009) found no significant direct association between neuroticism and the frequency of physical activity (including moderate and vigorous intensity exercises). Additionally, no other variables (including neuroticism) were significantly associated with the frequency of vigorous-intensity physical activity after controlling for sociodemographic factors or the five-factor personality domains. Possible reasons for this may include the limitations of measurement tools and sample size, or the potential interactive effects of individual health status, living environment, social support, and neuroticism, which were not included in the analysis and may have obscured the true relationship between physical activity and neuroticism. Campos-Uscanga et al. (2023) showed a negative correlation between neuroticism and total exercise duration among women, and a positive correlation between neuroticism and exercise frequency (days per week and minutes per day); however, this relationship was not confirmed in multivariate analyses. This may be because women with higher levels of neuroticism are more likely to increase exercise days in the short term to cope with emotional issues, but due to their emotional instability and susceptibility to negative emotions, they may struggle to maintain regular exercise habits in the long term, resulting in shorter exercise participation durations. Sale et al. (2000) did not find a significant association between neuroticism and physical activity, which may be due to the limitations of sample selection and the bias of self-reported physical activity. Moreover, the study did not measure the intensity of physical activity, focusing only on the frequency and duration of physical activity, which may have affected the significance of the results.

The meta-analysis of multiple regression also revealed the positive effect of a decrease in neuroticism on enhancing physical activity levels (average β = −0.150, *p* < 0.001). The findings of this study support the role of neuroticism in inhibiting individual physical activity, aligning with the conclusions of Kroencke et al. (2019). Individuals with lower neuroticism are more inclined to engage in and persist with physical exercise, as they typically exhibit greater emotional stability, which aids in better stress and challenge management, making it easier for them to maintain exercise routines and increase their physical activity levels. This perspective is supported by several studies. For instance, Satoh et al. (2022) found that individuals with lower levels of neuroticism are more likely to have better physical activity habits. Thøgersen-Ntoumani et al. (2022) discovered that individuals with higher neuroticism scores participated in physical activities with lower frequency and intensity. Similarly, Ahola et al. (2024) demonstrated that neuroticism is a significant factor influencing participation in physical and social activities.

The meta-analysis of multiple regression further revealed the positive effect of physical activity on reducing neuroticism (average β = −0.113). The study by Liao et al. (2022) found that physical activity may have a positive impact on neuroticism levels. Similarly, the research by Chan et al. (2018) indicated that individuals with higher levels of physical activity tend to have lower neuroticism scores, suggesting that they experience greater emotional stability with fewer negative emotions and emotional fluctuations. However, it is important to note that the meta-analysis showed a pooled effect size of −0.113 (95% CI: −0.273 to 0.047), and the confidence interval includes zero, indicating no significant overall effect of physical activity on neuroticism across the three studies. The significant statistical heterogeneity (*I*^2^ = 99.3%) and the non-significant effect sizes suggest that the slight negative correlation may not be practically significant. This might be due to the small number of included studies and measurement tool errors. Future research with larger sample sizes and more standardized measurement tools is needed to further investigate the relationship between physical activity and neuroticism.

Notably, the Finnish PASSWORD cohort's serial studies (Kekäläinen et al., 2020a,b, 2023; Ahola et al., 2024), using multidimensional approaches (e.g., combining accelerometer and self-report data), have revealed the dynamic moderating role of neuroticism on physical activity in middle-aged and older adults. Their results show: (1) The negative correlation between neuroticism and low-intensity leisure activities is significant in middle-aged women but weakens in older adults, indicating that age may moderate the impact of personality traits on exercise behavior; (2) Objective measurement tools (such as accelerometers) can more sensitively capture subtle correlations between neuroticism and physical activity levels, underscoring the value of multimethod integration in revealing complex mechanisms; (3) Cognitive function may mediate the negative relationship between neuroticism and physical activity (e.g., individuals with lower neuroticism may achieve greater cognitive improvements through exercise). While these findings enhance our understanding of the bidirectional relationship, the sample's focus on Nordic middle-aged and older adults may restrict the cross-cultural generalizability of the conclusions.

We acknowledge the limitations of our study, particularly regarding causality and study quality. The significant negative correlation between physical activity and neuroticism (average correlation coefficient *r* = −0.141) suggests that higher levels of neuroticism are associated with lower levels of physical activity. However, the small effect size indicates that while this relationship is statistically significant, it may not be practically significant in real-world applications. For instance, reducing neuroticism levels may only modestly increase physical activity levels. Therefore, we caution against over-interpreting the practical implications of this correlation. Future interventions should consider additional factors such as social support and environmental influences to enhance their effectiveness.

### Potential moderating roles of external factors

The qualitative findings of this review suggest that external factors such as gender and social support may moderate the relationship between neuroticism and physical activity. For instance, studies by Brunes et al. (2013) and Campos-Uscanga et al. (2023) consistently reported a stronger inhibitory effect of neuroticism on physical activity among females compared to males. This gender difference may be attributed to heightened emotional reactivity and greater susceptibility to social stressors in women. Furthermore, research by Saklofske et al. (2007) and Chan et al. (2018) indicates that social support may serve as a protective factor for individuals with high neuroticism, potentially buffering its negative impact by alleviating negative affect and enhancing motivation for physical activity engagement.

Despite these insights, the absence of systematic quantitative synthesis and formal moderation analyses precludes definitive conclusions regarding the magnitude and mechanisms of these moderating effects. Future studies should prioritize the collection and reporting of granular data on external factors to enable meta-regression or subgroup analyses, thereby advancing the understanding of their roles in the neuroticism–physical activity relationship.

### The hypothesized “negative feedback loop” between physical activity and neuroticism: underlying mechanisms

Our analysis revealed a significant negative correlation between physical activity and neuroticism, with neuroticism showing an inhibitory effect on physical activity (mean β = −0.150) and physical activity impacting neuroticism (mean β = −0.113). However, the meta-analysis of multiple regression indicates that the direct effect of physical activity on neuroticism is not statistically significant (overall effect size = −0.113, 95% CI: −0.273 to 0.047), and there is significant statistical heterogeneity (*I*^2^ = 99.3%). This lack of significance and high heterogeneity may stem from the limited number of studies (only three) and measurement tool errors. Yet, the data trend suggests physical activity might positively influence neuroticism levels. We propose a “negative feedback loop hypothesis,” where reducing neuroticism increases physical activity participation, which in turn lowers neuroticism. This hypothesis offers a direction for future research, which can validate the loop by increasing sample sizes and using more standardized measurement tools.

To better understand the relationship between neuroticism and physical activity, it is crucial to explore their underlying theoretical foundations and mechanisms. Psychologically, individuals with high neuroticism are prone to negative emotions like anxiety and depression, which can reduce their interest in physical activity. However, studies by Brunes et al. (2013) and Lochbaum et al. (2010) show that regular physical activity can lower neuroticism by enhancing emotion regulation and self-efficacy. Additionally, Satoh et al. (2022) found a negative correlation between physical activity and neuroticism, indicating a key role of social support. Biologically, physical activity boosts the release of neurotransmitters such as endorphins, dopamine, and serotonin, which are vital for emotion regulation and wellbeing (Cotman et al., 2007). Ahola et al. (2024) (noted a negative correlation between total physical activity volume and neuroticism levels. This may be because these neurotransmitters are released during physical activity, helping to regulate emotions. Desai et al. (2023) also mentioned that physical activity correlates with improved cognitive function, likely due to enhanced brain plasticity and reduced stress responses, which positively affects neuroticism levels.

In summary, the “negative feedback loop” hypothesis offers a comprehensive explanation of the relationship between physical activity and neuroticism. Despite its promise, the hypothesis requires further testing in future research with larger samples and more standardized measurement tools. Understanding the dynamic interplay and underlying mechanisms between physical activity and neuroticism is crucial for designing targeted psychological and behavioral interventions. Longitudinal and experimental studies are recommended to test this hypothesis and explore related moderators and mechanisms.

### Other associated factors in the improvement of neurotic personality through physical activity

The association between physical activity and neuroticism is influenced by a multitude of factors beyond the activity itself, including social support, individual experiences, gender, age, environmental factors, and the type of physical activity. Firstly, social support, a frequently cited key factor in the literature, plays a significant role in the engagement of physical activity and the level of neuroticism. Research by Saklofske et al. (2007) has shown that support from friends, family, and the community can increase the likelihood of individuals participating in physical activities. Furthermore, the study by Chan et al. (2018) suggests that social support can help neurotic individuals reduce anxiety and stress, thereby enhancing subjective wellbeing. Nakajima et al. (2023) also noted that individuals with lower subjective social status are more prone to depressive symptoms and may have higher levels of neuroticism, which may be related to resource access and social support. Individuals with lower social status may face greater life stressors, leading to increased neuroticism and a subsequent reduction in the willingness and ability to engage in physical activity. Secondly, the impact of individual experiences on the relationship between physical activity and neuroticism should not be overlooked. Sale et al. (2000) have indicated that individual experiences, such as athletic participation during childhood or experiencing trauma, may also affect the relationship between physical activity and neuroticism. Bessey (2018) further emphasized that individual experiences, such as childhood trauma or early life stress, may influence neuroticism levels and participation in physical activities.

Additionally, Campos-Uscanga et al. (2023) found in their study that the relationship between physical activity and neuroticism may be influenced by gender. Women may be more susceptible to the influence of neuroticism, exhibiting higher levels of negative emotions and neuroticism, which leads to lower duration and frequency of exercise participation. Nakajima et al. (2023) also discovered that women might be more prone to the impact of negative emotions and could face greater social pressures, resulting in increased neuroticism levels and a subsequent reduction in the willingness and ability to engage in physical activity. The research by Campos-Uscanga et al. (2023) suggests that, when considering multiple factors, neuroticism may not be a key factor influencing long-term physical activity in men.

Age is also an influential factor that cannot be overlooked. Adams and Nettle (2009) found that as people age, the frequency of their physical activity may decrease, which could be related to physiological capacity limitations, health issues, or lifestyle changes following retirement. The study by Brunes et al. (2013) also indicated that physical activity levels decline with age, while neuroticism levels may increase. However, Kekäläinen et al. (2020a) did not find a significant association between neuroticism and physical activity in older adults. This may be due to the limited range of physical activity levels in the elderly sample and the lower proportion of participants engaging in regular physical exercise and sports. The study also revealed that elderly individuals with higher neuroticism reported less physical activity compared to accelerometer assessments, which could be attributed to the influence of neuroticism on their perception of their own activity levels.

Furthermore, different types and intensities of physical activity may have varying impacts on neuroticism. Nakajima et al. (2023) indicated in their study that higher levels of physical activity are associated with lower neuroticism, and neuroticism may mediate the relationship between physical activity and depressive symptoms, suggesting that physical activity could reduce depressive symptoms by lowering neuroticism levels. Desai et al. (2023) also found that as neuroticism levels increase, there is a significant decline in vigorous, moderate, and overall physical activity levels, with an increase in sedentary time. The results of Tsartsapakis et al. (2023) showed that individuals engaged in outdoor fitness activities exhibited lower neuroticism levels compared to those participating in indoor fitness activities. This may be related to the additional psychological benefits provided by the outdoor environment, such as stress reduction, increased wellbeing, and emotional recovery. However, the study by Wilson et al. (2015) suggests that in the young female population, individuals with higher neuroticism may have lower levels of physical activity. Yet, the study did not find a significant association between neuroticism and MVPA, which differs from other research findings and may reflect variations in the impact of neuroticism on physical activity intensity across different study samples or measurement methods. Additionally, Adams and Nettle (2009) found no significant correlation between neuroticism and the frequency of moderate-intensity physical activity after controlling for sociodemographic and personality domain factors (such as conscientiousness), which could be due to biases in self-reported physical activity data.

Lastly, some of this study's samples are from Nordic countries (like Finland and Norway), whose high-latitude geography causes marked seasonal climate changes, such as long winters and insufficient sunlight. Such surroundings may influence the relationship between neuroticism and physical activity through the following ways:First, the lack of winter sunlight may cause seasonal affective disorder (SAD), which can worsen anxiety or depression, as Rosenthal et al. (1984) found. Second, extremely cold weather may restrict outdoor exercise options, as Kekäläinen et al. (2020a) pointed out, pushing people toward indoor activities. These activities, due to their weak psychological benefits from lack of natural environment ties (Tsartsapakis et al., 2024, 2023), have a weaker effect on emotion regulation. These environmental factors might either overstate the negative correlation between neuroticism and physical activity in Nordic samples or mask the role of social support and other moderating mechanisms. Future research should properly document climate and latitude variables and use cross-regional comparisons (like between Nordic countries and low-latitude areas) to check the ecological validity of results. A limitation of this study is that it failed to control for geographical and climatic factors like seasonal light changes, which might affect the conclusion's validity and generalizability across different environments.

### The heterogeneity of neuroticism facets in relation to physical activity

In the 25 included studies, some measured and analyzed different aspects of neuroticism. We explored the heterogeneity from these aspects as follows. Differences in neuroticism measurement existed across studies. Some used only the total neuroticism score, while others distinguished its main facets, such as anxiety, impulsivity, and vulnerability. These measurement differences may lead to inconsistencies in the relationship between neuroticism and physical activity. For example, Brunes et al. (2013) found a negative correlation between overall neuroticism and physical activity, but in subdimension analyses, impulsivity showed a more significant association. This suggests that focusing solely on the total score may mask differences in the associations of neuroticism's subdimensions with physical activity. Most studies found that neuroticism significantly inhibits physical activity, but the extent varies across facets. Impulsivity may more substantially reduce physical activity frequency and duration due to difficulty in adhering to-term long exercise plans (e.g., Ahola et al., 2024), while anxiety may more affect the willingness to engage in high-intensity physical activity (e.g., Brunes et al., 2013). Heterogeneity also exists in the impact of physical activity on neuroticism. Some studies found that only high-intensity physical activity significantly alleviates anxiety and depressive symptoms within neuroticism (e.g., Satoh et al., 2022), with smaller effects on other facets like impulsivity. This indicates that the beneficial effects of physical activity on neuroticism may be selective, varying in degree across different facets.

When exploring the relationship between physical activity and neuroticism, this study found that the major facets of neuroticism (such as anxiety, impulsivity, and vulnerability) showed heterogeneous relationships with physical activity. This may be due to the intrinsic differences among these facets. Anxiety, marked by excessive worry and tension in response to external stimuli, can affect an individual's motivation and experience of physical activity. Impulsivity, related to self-control and decision-making, impacts the persistence and execution of physical activity plans. Vulnerability, associated with self-worth and stress-coping ability, may indirectly influence physical activity participation. Previous studies have used different definitions and measurement tools for neuroticism, which may have hindered our ability to fully capture the unique roles of each facet. While some studies employed comprehensive tools like the NEO-PI to assess multiple facets of neuroticism, others used more concise tools like the BFI, which provides a more general measure. Future research on physical activity and neuroticism should focus more on differentiating and analyzing the facets of neuroticism. On the one hand, we need to explore the unique mechanisms through which each facet is linked to physical activity, such as anxiety being alleviated via cognitive reappraisal in physical activity, and impulsivity being improved through self-control training. On the other hand, it is essential to standardize neuroticism measurement by using multidimensional tools that cover its main facets to enhance result accuracy and comparability. Additionally, future research should investigate the interactions among neuroticism facets and their combined impact on physical activity and health.

Several methodological considerations should be noted. First, although the conversion of Spearman ρ to Pearson *r* followed established approximations, non-linear relationships may bias the pooled estimates and warrant cautious interpretation. Second, despite subgroup analyses by age and assessment tool, residual heterogeneity remained high (*I*^2^ > 90 %), suggesting unmeasured moderators such as neuroticism facets (anxiety, impulsivity), cultural context, or climatic factors. Finally, Egger's test (*P* > 0.05) and symmetrical funnel plots indicated no evidence of publication bias, so trim-and-fill was not applied; nevertheless, the small number of studies (*n* = 25) limits the power to detect small-study effects. These issues are explicitly addressed in the following limitations section.

### Clinical and practical implications

Our study reveals a significant negative association between neuroticism and physical activity, suggesting that higher neuroticism levels are linked to lower physical activity. This finding underscores the potential of physical activity as a strategy for improving mental health. Mental health professionals can incorporate physical activity recommendations into treatment plans for individuals with high neuroticism, emphasizing the benefits of exercise for mood regulation and stress reduction. Given the modest effect size, interventions should be personalized, considering additional factors such as social support and environmental influences. Public health initiatives should also target individuals with high neuroticism by addressing common barriers to physical activity. Future research should explore the underlying mechanisms and develop interventions that specifically address the impact of neuroticism on physical activity.

In conclusion, our findings highlight the need for personalized and multifaceted interventions to effectively address the complex interplay between neuroticism and physical activity. By integrating physical activity recommendations into mental health treatments and public health campaigns, we can potentially improve mental health outcomes and promote healthier lifestyles.

### Limitations and recommendations

This study has several limitations that warrant careful interpretation. First, the predominance of cross-sectional designs restricts causal inferences regarding the bidirectional relationship between neuroticism and physical activity, as the limited number of longitudinal studies (*n* = 8) and scarcity of intervention research preclude robust exploration of temporal dynamics or causality. Second, substantial heterogeneity (*I*^2^ > 90% in subgroup analyses) persists despite stratification by age and assessment tool, potentially stemming from unmeasured moderators such as cultural context, gender, neuroticism subdimensions, or reliance on self-reported measures, which may introduce variability; while accelerometer-based studies were included (*n* = 3), their small sample size limits precision. Third, excluding non-English studies and small samples (*n* < 50) may introduce selection and language bias, risking the oversight of culturally unique findings, particularly from low- and middle-income countries. Fourth, although funnel plots and Egger's test indicated no significant publication bias, the small study count (*n* = 25) limits power to detect “file drawer effects” (unpublished null results), and complementary methods like trim-and-fill analysis or gray literature searches are recommended to mitigate this risk. Fifth, sensitivity analyses (e.g., excluding low-quality or self-report–only studies) were not fully conducted, and while preliminary checks suggested robustness, formal analyses are needed to quantify impacts. Finally, seasonal or climatic factors were not accounted for, which may confound results in high-latitude regions. Future research should address these gaps through longitudinal and experimental designs, standardized assessments, meta-regression to explore heterogeneity sources, inclusion of multilingual studies with sensitivity analyses, and documentation of environmental variables to enhance ecological validity.

## Conclusion

Our study confirms a significant yet modest negative association between neuroticism and physical activity, suggesting higher neuroticism levels are linked to lower physical activity. Given the small effect size, interventions targeting neuroticism should incorporate additional factors like social support and environmental influences for enhanced efficacy. Future research must employ longitudinal and experimental designs, standardized objective measures, and diverse samples to establish causality, explore bidirectional dynamics, and mitigate potential biases such as the “file drawer effect.” While our findings highlight physical activity's potential role in improving mental health, they also underscore the need for rigorous methodologies to fully understand this complex relationship.

## Data Availability

The original contributions presented in the study are included in the article/Supplementary material, further inquiries can be directed to the corresponding authors.
