# Correction: Association between neuroticism and physical activity: a systematic review and meta-analysis

**DOI:** 10.3389/fnhum.2025.1713272

**Published:** 2025-10-09

**Authors:** Wenxue Ma, Xiaotian Wang, Wentao Qiu, Yuyang Nie, Rong Gao, Cong Liu

**Affiliations:** ^1^College of Physical Education and Sports, Beijing Normal University, Beijing, China; ^2^College of Education for the Future, Beijing Normal University, Zhuhai, China

**Keywords:** neuroticism, personality, physical activity, correlation, influencing factors, potential psychological mechanisms

The order of authors in the author list of the published paper was erroneously given as:

Rong Gao, Cong Liu, Yuyang Nie, Wentao Qiu, Xiaotian Wang, Wenxue Ma

The correct author list reads:

Wenxue Ma^†^, Xiaotian Wang^†^, Wentao Qiu, Yuyang Nie, Rong Gao^*^, Cong Liu^*^

^†^These authors have contributed equally to this work

The author contributions statement was erroneously given as RG: Conceptualization, Formal analysis, Methodology, Project administration, Resources, Supervision, Visualization, Writing – original draft, Writing – review & editing. CL: Formal analysis, Funding acquisition, Methodology, Project administration, Resources, Software, Supervision, Validation, Writing – original draft, Writing – review & editing. YN: Formal analysis, Validation, Writing – original draft. WQ: Data curation, Methodology, Visualization, Writing – original draft. XW: Data curation, Methodology, Project administration, Writing – review & editing. WM: Conceptualization, Formal analysis, Investigation, Software, Validation, Writing – original draft, Writing – review & editing.

The correct author contributions statement is WM: Conceptualization, Formal analysis, Investigation, Software, Validation, Writing – original draft, Writing – review & editing. XW: Data curation, Methodology, Project administration, Writing – review & editing. WQ: Data curation, Methodology, Visualization, Writing – original draft. YN: Formal analysis, Validation, Writing – original draft. RG: Conceptualization, Formal analysis, Methodology, Project administration, Resources, Supervision, Visualization, Writing – original draft, Writing – review & editing. CL: Formal analysis, Funding acquisition, Methodology, Project administration, Resources, Software, Supervision, Validation, Writing – original draft, Writing – review & editing.

The original version of this article has been updated.

